# The mechanosensory channel PIEZO1 functions upstream of angiopoietin/TIE/FOXO1 signaling in lymphatic development

**DOI:** 10.1172/JCI176577

**Published:** 2024-05-15

**Authors:** Jing Du, Pan Liu, Yalu Zhou, Sol Misener, Isha Sharma, Phoebe Leeaw, Benjamin R. Thomson, Jing Jin, Susan E. Quaggin

**Affiliations:** 1 Feinberg Cardiovascular and Renal Research Institute,; 2 Department of Ophthalmology, and; 3 Division of Nephrology, Department of Medicine, Northwestern University, Feinberg School of Medicine, Chicago, Illinois, USA.

**Keywords:** Development, Vascular biology, Embryonic development, Endothelial cells, Ion channels

## Abstract

Lymphedema is a debilitating disease with no effective cure and affects an estimated 250 million individuals worldwide. Prior studies have identified mutations in piezo-type mechanosensitive ion channel component 1 (*PIEZO1*), angiopoietin 2 (*ANGPT2*), and tyrosine kinase with Ig-like and EGF-like domains 1 (*TIE1*) in patients with primary lymphedema. Here, we identified crosstalk between these molecules and showed that activation of the mechanosensory channel PIEZO1 in lymphatic endothelial cells (LECs) caused rapid exocytosis of the TIE ligand ANGPT2, ectodomain shedding of TIE1 by disintegrin and metalloproteinase domain–containing protein 17 (ADAM17), and increased TIE/PI3K/AKT signaling, followed by nuclear export of the transcription factor FOXO1. These data establish a functional network between lymphedema-associated genes and provide what we believe to be the first molecular mechanism bridging channel function with vascular signaling and intracellular events culminating in transcriptional regulation of genes expressed in LECs. Our study provides insights into the regulation of lymphatic function and molecular pathways involved in human disease.

## Abstract 2

The mechanosensory channel PIEZO1 triggers an ANGPT/TIE/FOXO1 signaling pathway in the mouse lymphatic system.

## Graphical abstract

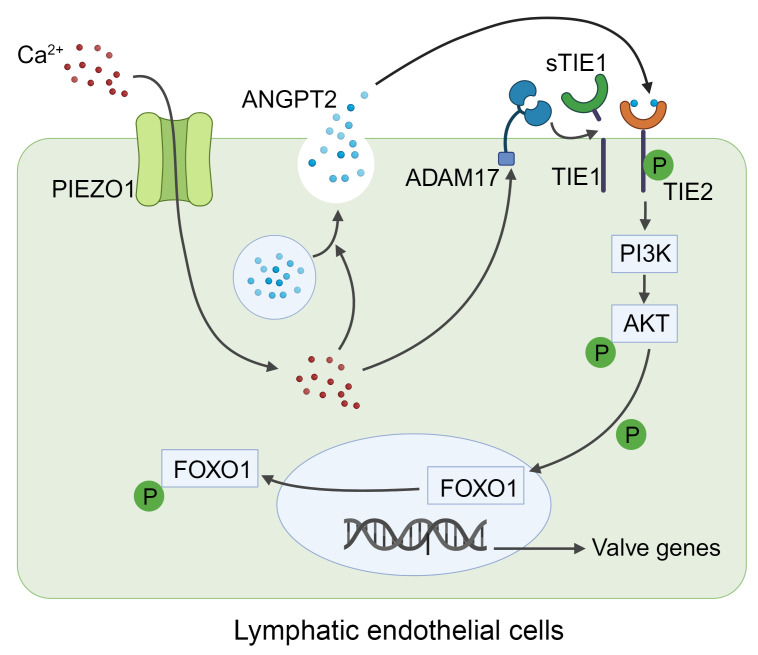

## Introduction

Lymphedema is a common disease worldwide, with no cure and variable response to interventions. It may occur as the result of mutations in genes involved in lymphatic development or can be acquired, especially in patients following breast cancer surgery. Despite recent exciting advances in the lymphatic field, our understanding of mechanisms regulating the development and function of the lymphatic circulation is incomplete. Further understanding of signals and pathways that regulate lymphatic endothelial cell (LEC) development is urgently needed to develop new therapies for lymphedema and other lymphatic disorders. In this study, we identified what we believe to be a previously unknown molecular mechanism linking a nonselective mechanosensory cationic channel and a lymphatic vascular tyrosine kinase signaling pathway. Unlike the blood vasculature, lymphatic vessels are not subject to high-pressure flow but instead experience low-pressure laminar and oscillatory flow cues (1). Furthermore, sprouting of lymphatic tip cells may occur under static conditions downstream of cellular responses to these mechanical and other local environmental forces, which is critical for normal development and maintenance of lymphatic vessels and valves. Loss-of-function mutations in the gene piezo-type mechanosensitive ion channel component 1 (*PIEZO1*), which encodes a mechanosensory nonselective cation channel triggered by oscillatory shear stress (OSS) or mechanical stretch, cause lymphatic valve and vessel defects resulting in lymphedema in mice and humans, underscoring the importance of physical cues for lymphatic integrity (2–7). In keeping with this, OSS or PIEZO1 activation modeled in vitro can regulate key lymphatic genes such as *FOXC2*, *GATA2*, *GJA4*, and *ITGA9* (3, 8, 9).

In addition to mechanical forces, 2 vascular tyrosine kinase receptor signaling pathways are critical for the development of the lymphatic circulation in mice and humans: the VEGFC/VEGFR3 and the angiopoietin/TIE pathways. In the lymphatic system, VEGFR3/FLT4 and TIE receptors are predominantly expressed by LECs, whereas the VEGFR3 ligand VEGFC is produced by macrophages, and the angiopoietin 2 (ANGPT2) ligand is expressed by and stored in vesicles in LECs themselves.

In mice, genetic deletion of *Vegfc* or *Flt4* (*Vegfr3*) results in a complete absence of lymphatic development from the earliest budding of LECs from the cardinal vein (10–13). By contrast, deletion of tyrosine kinase with Ig-like and EGF-like domains 1 (*Tie1*) or *Angpt2* results in later — albeit profound — defects in lymphatic development including failure of collecting vessel and lymphatic valve formation, nuchal edema, and chylous ascites due to abnormal morphology and patterning of lymphatic capillaries and lymphatic sacs (14–17). Interestingly, although the ANGPT1 ligand can substitute for ANGPT2 function in the lymphatics, as shown by rescue of the lymphatic phenotype in transgenic mice expressing a cDNA for *Angpt1* in the *Angpt2* locus, it is unlikely to function as an endogenous ligand responsible for lymphatic development, given the lack of expression in developing lymphatic vessels (17). As TIE1 is reported to function as an orphan receptor and does not bind to ANGPT2 directly, current models support a role for cross-regulation of the TIE receptors to transduce the ligand-dependent intracellular signals needed for lymphatic formation (18–20). While TIE1 is the dominant receptor for lymphatic development, *Tie2*-deficient mice also exhibit defects in lymphatic vessel and valve formation (16, 18, 21–23). Recent work showed TIE1-regulated cell-surface expression of VEGFR3 (FLT4) in lymphatic endothelium, demonstrating crosstalk between these 2 growth factor pathways (23). In humans, similar to *PIEZO1*, mutations in *FLT4*, *ANGPT2*, and *TIE1* have been associated with lymphedema, underscoring the conservation of function across species (6, 7, 24, 25).

The essential role for TIE1 during LEC remodeling and dynamic shape change, such as lymphatic valve formation, that are known to occur downstream of mechanical signals, and the fact that TIE1 expression is upregulated at sites experiencing OSS and mechanical stimulation such as at bifurcations of blood and lymphatic vessels, led us to speculate that TIE1 function must be linked to a mechanical and/or extracellular signal(s) at the molecular level (26–28). To test this hypothesis, we used a combination of animal models, RNA-Seq analysis, and biochemical approaches. Our data showed that the mechanically activated channel PIEZO1 functions upstream of TIE1 and ANGPT2/TIE signaling in the lymphatics. Together, our study reports what to our knowledge is the first molecular network linking mechanical and cation-stimulated signals to intracellular signaling events that involve proteins known to be important for lymphatic function and disease in patients.

## Results

### TIE1 is required for lymphatic vessel and valve development.

**Figure 1:**
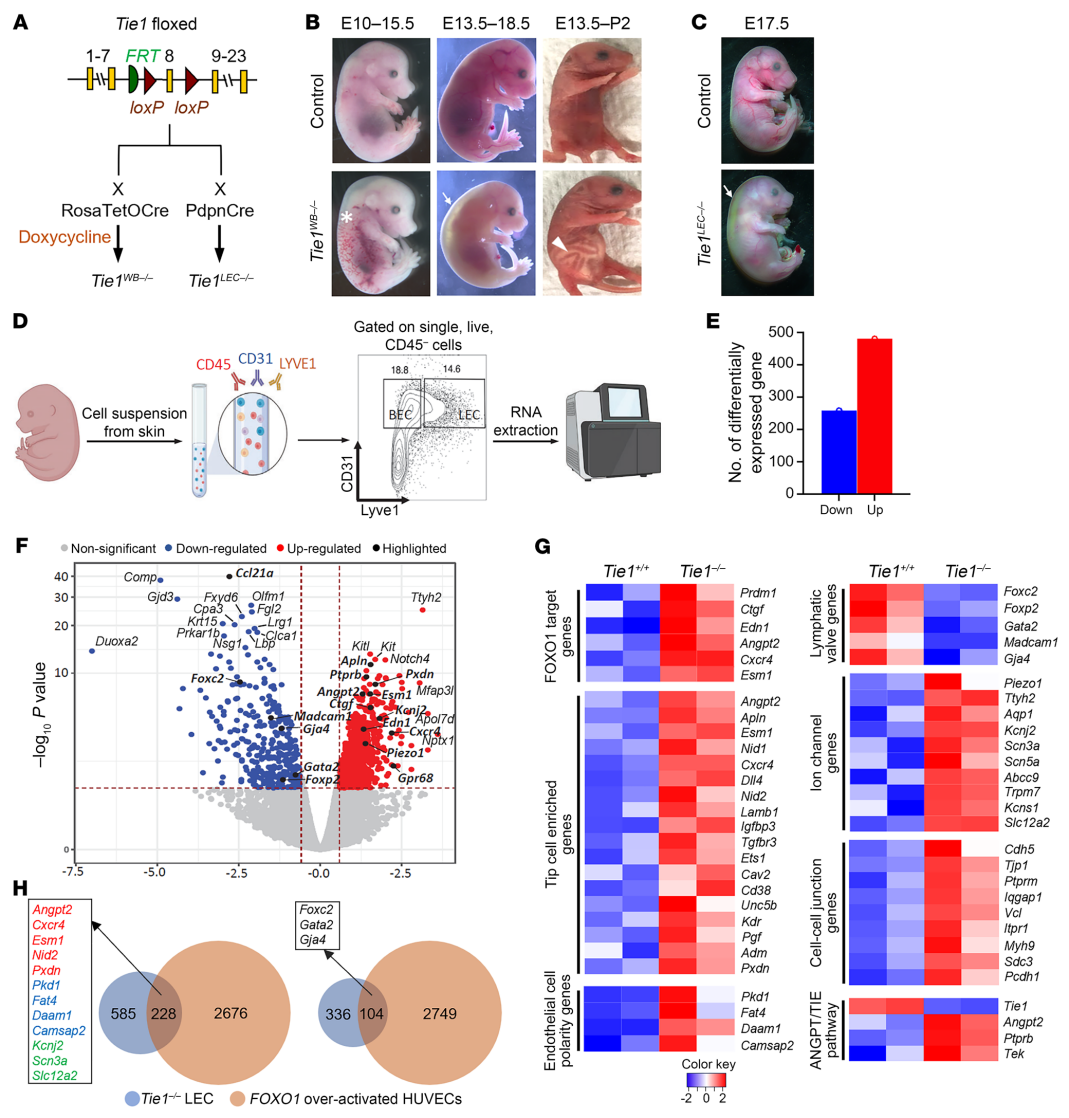
Bulk RNA-Seq analysis of dermal LECs reveals transcriptomic changes driven by FOXO1 overactivation in the *Tie1^–/–^* group. (**A**) Generation of *Tie1* whole-body inducible or lymphatic endothelium–specific *Tie1*-KO mice. *Tie1* whole-body inducible KO (*Tie1^WB–/–^*) was generated by crossing *Tie1^fl^* mice with *Rosa26*^rtTA^ TetOCre mice and timed induction with Dox water. *Tie1* lymphatic-specific *Tie1* KO (*Tie1^LEC–/–^*) was generated by crossing *Tie1^fl^* mice with *Pdpn*Cre mice. (**B**) Gross phenotypes of *Tie1^WB–/–^* embryos that were induced and examined at different time points (first number indicates the induction time point; second number indicates the harvest time point). White asterisk shows blood-filled lymphatics at E15.5 (5 of 7 of the *Tie1^WB–/–^* embryos); arrow shows edema at E18.5 (7 of 9 of the *Tie1^WB–/–^* embryos); and arrowhead shows chylous ascites at P2 (6 of 6 of the *Tie1^WB–/–^* pups). (**C**) LEC-specific *Tie1* KO resulted in a phenotype similar to that of whole-body KO. (**D**) Workflow of dermal LEC bulk RNA-Seq. E18.5 embryos induced at E13.5 were euthanized. The skin from each embryo was removed and placed in an Eppendorf tube for enzymatic digestion. The single-cell suspension was labeled with antibodies against CD45, CD31, and Lyve1. CD31^+^LYVE1^+^ cells were sorted into lysis buffer for RNA extraction. Bulk RNA-Seq was performed on the Illumina HiSeq 4000 system. (**E**) Number of differentially expressed genes using a *P* value of less than 0.01 as the cutoff. Down, downregulated; Up, upregulated. (**F**) Volcano plot shows some of the most differentially expressed genes including *Ccl21a*, valve genes and tip cell–enriched genes. (**G**) Heatmaps of manually selected vascular-relevant genes categorized as labeled. (**H**) Overlap between our data set (blue) and a data set from HUVECs with transcriptionally active FOXO1 (orange). Some commonly regulated genes are listed in the square, including tip cell genes (red), polarization genes (blue), ion channel genes (green), and valve genes (black).

Given the central role of ANGPT/TIE signaling and especially the central role of TIE1 in lymphatic development, we generated several mouse models of *Tie1* deletion using an inducible, whole-body Cre deleter system (*Rosa26*^rtTA^ TetOCre) as well as a lymphatic-selective Cre deleter (podoplanin-Cre [*Pdpn*Cre]). We confirmed that our model recapitulated previously published reports and identified new phenotypes in the lymph nodes (Figure 1, our unpublished observations, and Supplemental Figure 1; supplemental material available online with this article; https://doi.org/10.1172/JCI176577DS1). Timed *Tie1* deletion was accomplished by providing female mice with doxycycline (Dox) water at different gestational time points (Figure 1A). The deletion was confirmed by whole-mount skin TIE1 immunostaining (Supplemental Figure 1A). *Tie1* deletion at E10.5 (*Tie1^WB–/–E10.5^*) resulted in edema and blood-filled lymphatic vessels visible at E15.5 (Figure 1B, asterisk) and intrauterine embryonic lethality at approximately E18.5. *Tie1^WB–/–E13.5^* mice displayed severe subcutaneous and nuchal edema at E18.5 (Figure 1B, arrow) and chylous ascites at P2 (Figure 1B, arrowhead). Similarly, lymphatic-specific *Tie1* deletion (*Tie1^LEC–/–^*) (Figure 1A) using the *Pdpn*Cre driver (29) resulted in severe subcutaneous edema at E17.5 (Figure 1C arrow), confirming a lymphatic-specific requirement for TIE1. Whole-mount immunostaining of embryonic dorsal skin with the lymphatic marker PROX1 showed reduced expansion of developing lymphatic vessels in *Tie1^WB–/–E11.5^* mice at E16.5 (Supplemental Figure 1B). Analysis of the lymph sacs at E15.5 showed expression of the lymphatic markers LYVE1 and PROX1 in control mice and mutant mice, however, *Tie1^WB–/–E10.5^* mice lacked lymphovenous valves (Supplemental Figure 1C, arrow). At E16.5, we found that the dermal lymphatic vessels were completely devoid of lymphatic valves that could be identified as PROX1^hi^ LEC clusters in control mice compared with *Tie1^WB–/–E13.5^* mice (Supplemental Figure 1D). Similarly, we observed a complete failure of lymphatic valve development in the mesentery at E18.5 in mutant mice (Supplemental Figure 1E). VEGFR3/FLT4 and PROX1 staining of dermal lymphatics was seen in both mutant and WT lymphatics but appeared at lower intensity in mutants, and was associated with abnormal lymphatic patterning with irregular diameters and bulging regions (Supplemental Figure 1D).

### RNA-Seq analysis of dermal LECs identified differential gene expression between Tie1^+/+^ and Tie1^–/–^ LECs.

To better understand the molecular mechanism(s) underlying the lymphatic phenotype following *Tie1* deletion, we performed bulk RNA-Seq on FACS-sorted E18.5 dermal LECs (Figure 1D) that were induced at E13.5. The embryos were divided into *Tie1*^+/+^ and *Tie1^–/–^* groups on the basis of the genotyping results. Using the statistical cutoff of an adjusted *P* value of less than 0.01, we identified 260 downregulated and 482 upregulated genes (Figure 1E). We validated the RNA-Seq results with skin whole-mount immunostaining and human dermal LEC (HDLEC) quantitative reverse transcription PCR (qRT-PCR) for several selected genes including *Foxc2*, *Cdh5*, *Piezo1*, *Angpt2*, and *Prdm1* (Supplemental Figure 2, A and B). The results were consistent with RNA-Seq, confirming the robustness of the RNA-Seq data. A volcano plot highlights the most differentially expressed genes identified in *Tie1*-deficient mice compared with control mice (Figure 1F). Importantly, many genes that are involved in lymphatic valve formation were downregulated in *Tie1^–/–^* LECs, including *Foxc2*, *Gja4*, *Gata2*, *Foxp2*, and *Madcam1*, suggesting that a shared pathway by these genes regulates lymphatic valve development. Among the most upregulated genes, we identified a group of tip cell–enriched genes including *Apln*, *Angpt2*, *Esm1*, and *Cxcr4* (Figure 1G and Supplemental Figure 2B).

### RNA-Seq analysis of dermal LECs suggests regulation of a FOXO1-mediated mechanism.

Given that TIE1 is a component of a growth factor signaling pathway, we next looked for a transcription factor that might govern the observed transcriptional changes. From the RNA-Seq data, we observed that several classic FOXO1 target genes, including *Ctgf*, *Edn1*, *Angpt2*, *Cxcr4*, and *Esm1*, were upregulated in the *Tie1* mutants (Figure 1G). The recently identified FOXO1 target gene *Prdm1* (30) was also upregulated in *Tie1*^–/–^ LECs. These data suggested that TIE1 may regulate lymphatic development and function by modulating FOXO1 activity. Therefore, we performed overlapping analysis between our *Tie1* data set and a published data set from human umbilical vein ECs (HUVECs) expressing an activated version of FOXO1 (31). There were substantial similarities in both up- and downregulated gene categories between the 2 data sets (Figure 1H). In *Tie1*-deficient mice, we also observed an intriguing phenomenon that supports a role for increased activity of FOXO1 in LECs. In the lymphatic plexus of mutant mice, many LEC nuclei were observed at the periphery of the vessel, while they were evenly distributed in the control mice (Supplemental Figure 3, A and B). These features suggested a “hyperpolarized phenotype,” which we confirmed by measurements of the Golgi-nuclear angle (Supplemental Figure 3, C and D), a phenotype known to be dependent on FOXO1 activity (32, 33).

### TIE1 and ANGPT/TIE activation regulate FOXO1 subcellular localization in vivo and in vitro.

**Figure 2:**
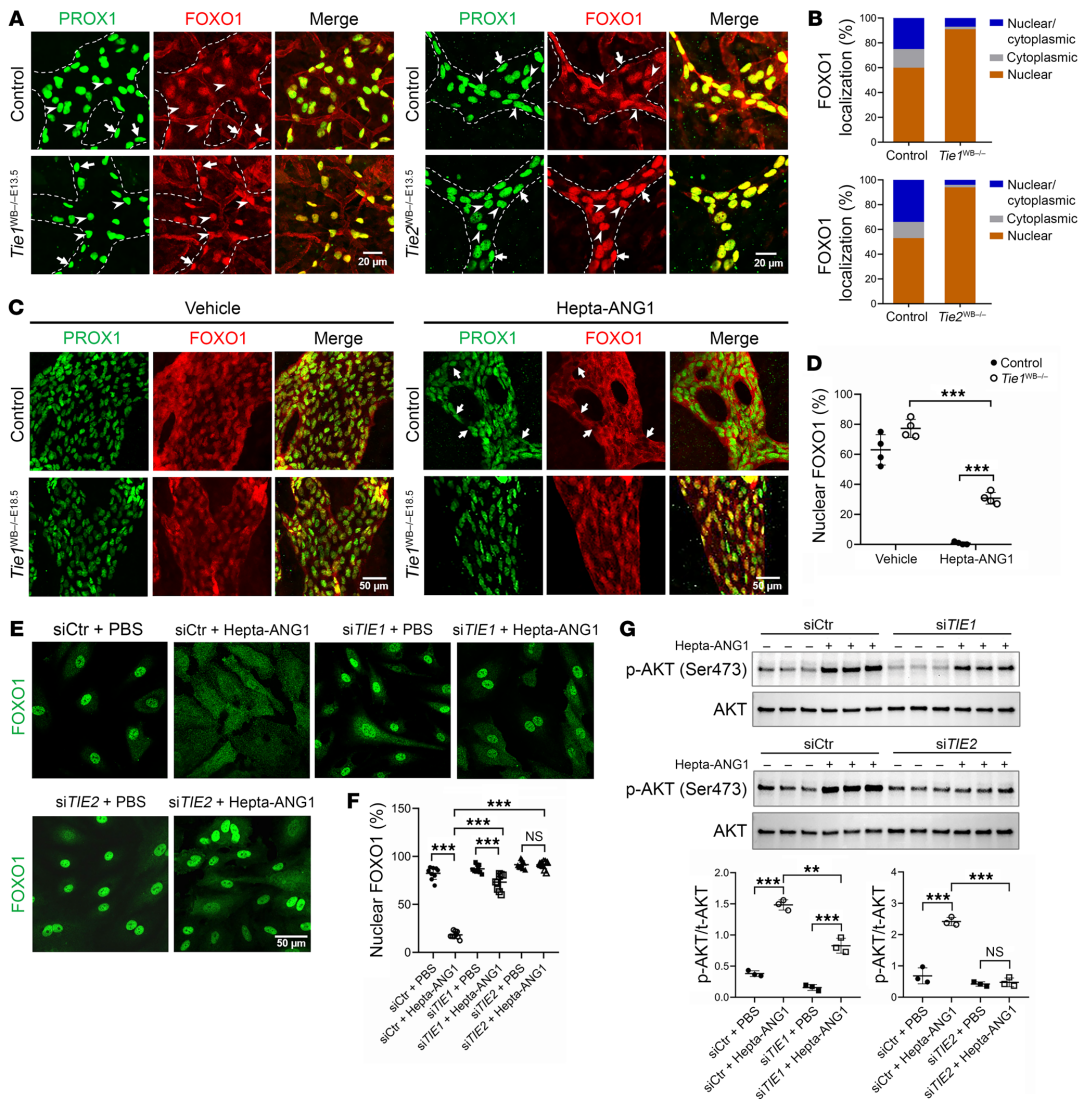
TIE-mediated regulation of FOXO1 subcellular localization in vivo and in vitro. (**A**) Whole mounts of E16.5 skin with lymphatic vessels stained for PROX1 and FOXO1. Nuclei within areas of LECs from WT control, *Tie1^WB–/–E13.5^*, and *Tie2^WB–/–E13.5^* mice are indicated by arrowheads (branching points) and arrows (nonbranching points). Scale bars: 20 μm. (**B**) Quantification of FOXO1 localizations in PROX1^+^ LECs. Averaged values calculated from 3 mice in each group are presented. (**C**) P1 *Tie1^WB–/–E18.5^* pups and their littermate controls were intraperitoneally injected with 1 μg/g BW Hepta-ANG1 or PBS. After a 30-minute period, pups were euthanized, and mesentery specimens were harvested and stained for PROX1 and FOXO1. Scale bars: 50 μm. (**D**) Quantification of FOXO1 localizations from 4 mice in each group. (**E**) FOXO1 staining of HDLECs transfected with siCtr, si*TIE1*, or si*TIE2* for 48 hours and subsequently treated with Hepta-ANG1 (1 μg/mL) or PBS for 30 minutes. This experiment was replicated 3 times. Scale bar: 50 μm. (**F**) Quantification of FOXO1 localization in 3 fields of view selected from each group. (**G**) Western blot analysis of p-AKT levels in HDLECs transfected with siCtr, si*TIE1*, or si*TIE2* and treated with either vehicle or Hepta-ANG1. Each band represents a biological replicate sample (*n* = 3). Data are expressed as the mean ± SD. **P < 0.01 and ***P < 0.001, by 2-tailed, unpaired Student’s *t* test (**D**) and 2-way ANOVA followed by Tukey’s multiple-comparison test (**F** and **G**).

To examine whether there were changes in FOXO1 protein expression in vivo, which could explain the RNA-Seq data and the observed overlap with FOXO1-regulated RNA-Seq data sets, we stained the skin of mice using whole-mount immunohistochemistry and antibodies against FOXO1 and PROX1. The fluorescence intensity of FOXO1 was comparable between control and *Tie1^WB–/–E13.5^* mice in the lymphatic vessels (Figure 2A), suggesting that *Tie1* deletion did not change FOXO1 at the level of transcription or protein production. Indeed, in the RNA-Seq data from *Tie1*-deficient mice, *Foxo1* transcript expression was unchanged. Since FOXO1 signaling can be dynamically regulated by shuttling of FOXO1 from the nucleus to the cytosol, we next analyzed whether the localization of FOXO1 in relation to PROX1-labeled LEC nuclei was changed in the mutants. In the control group, LECs in the nonbranching areas displayed mainly nuclear staining of FOXO1. However, in areas of vessel branching, we found that LEC nuclear FOXO1 staining was decreased and cytosolic staining was increased, probably due to a mechanical force change at the vessel branch points, which triggers the translocation of FOXO1 (30, 34). In contrast, in *Tie1^WB–/–E13.5^* mice, FOXO1 staining was mainly confined to the nucleus throughout the vessel, even at branch points (arrowheads) and nonbranched areas (Figure 2, A and B). This result supported a model in which TIE1 is required for the translocation of FOXO1 from the nucleus to the cytosol, thus modulating FOXO1 activity. Since TIE1 serves as a functional modulator of ANGPT/TIE2 signaling, we further explored the alteration in FOXO1 localization within *Tie2^WB–/–E13.5^* LECs as well as LEC expression of TIE1, TIE2, ANGPT2, and FOXO1 in *Tie1*-deficient and WT embryos and/or pups. Similar to our observations in *Tie1*^WB–/–E13.5^ mice, the results revealed that FOXO1 in *Tie2^WB–/–E13.5^* LECs exhibited exclusive localization within the nucleus (Figure 2, A and B). Immunostaining confirmed the deletion of TIE2 from LECs in *Tie2-*mutant embryos (Supplemental Figure 4).

Next, we costained mesenteric lymphatic vessels for TIE1, TIE2, and ANGPT2, which showed broad expression of both TIE receptors in WT LECs at E16.5 that was lost in *Tie1-*mutant E16.5 embryos (Supplemental Figure 5). At this time point, ANGPT2 was not detectable in WT lymphatic vessels but was observed in TIE1-deficient lymphatics (Supplemental Figure 6A), suggesting intracellular accumulation of the ligand. By P1 and P5, TIE1 and ANGPT2 were more highly expressed in lymphatic valve–forming regions in WT mice, whereas TIE2 expression remained more homogenously distributed (Supplemental Figure 5 and Supplemental Figure 6B). As predicted, the ratio of cytosolic/nuclear localization of FOXO1 was increased in valve regions, consistent with a requirement for a transient reduction in FOXO1 activity for lymphatic valve formation (Supplemental Figure 6B). The postnatal *Tie1*-mutant mice did not survive in the perinatal period, thus precluding a comparison with WT LECs at these time points.

### TIE-mediated nuclear translocation of FOXO1 is ligand dependent.

To further support a role for TIE1 and ANGPT/TIE signaling in FOXO1 translocation in LECs, we injected an ANGPT1 mimic, Hepta-ANG1 (35), intraperitoneally into P1 pups and stained the mesentery to examine FOXO1 localization. Hepta-ANG1 is a strong stimulator of TIE2/TEK activation (35). Thirty minutes after Hepta-ANG1 injection, in the control mesentery, the majority of the FOXO1 staining was excluded from the nuclei, even in nonbranching areas, whereas in the vehicle-injected group, we observed FOXO1 staining mainly in the nuclei. However, in *Tie1* mutants, Hepta-ANG1 did not result in nuclear extrusion of FOXO1 as effectively, resulting in a mosaic FOXO1 pattern (Figure 2, C and D). This result further supports a model in which TIE1 is required for ligand-dependent FOXO1 shuttling in lymphatic endothelium.

In cultured cells, we observed a similar pattern of FOXO1 localization in Hepta-ANG1–treated HDLECs. In control siRNA–treated (siCtr-treated) HDLECs, we found that Hepta-ANG1 effectively removed FOXO1 from the nucleus to the cytoplasm. However, in si*TIE1*- or si*TIE2*-treated HDLECs, the ability of Hepta-ANG1 to remove FOXO1 was largely reduced (Figure 2, E and F). Because AKT activation is known to regulate FOXO1 trafficking, we analyzed whether TIE1 and TIE2 also modulated AKT activation. Indeed, we found that Hepta-ANG1 treatment significantly increased phosphorylated AKT (p-AKT) levels in control HDLECs and phosphorylation of TIE receptors (Figure 2G and Supplemental Figure 7, A and B). However, si*TIE1* treatment blunted and si*TIE2* treatment abolished the Hepta-ANG1–induced AKT phosphorylation in HDLECs (Figure 2G). Together, these data from in vivo and in vitro assays suggested that both TIE1 and TIE2 modulated AKT phosphorylation and regulated FOXO1 signaling in LECs and that these effects were ligand dependent.

### Tie1-deficient LECs exhibit increased expression of ion channels.

In addition to the identification of FOXO1 target gene alteration, another category of genes that were upregulated in the mutants involved those encoding ion channels, including *Piezo1*, *Ttyh2*, *Aqp1*, *Kcnj2*, *Scn3a*, *Scn5a*, *Abcc9*, *Trpm7*, *Kcns1*, and *Slc12a2* (Figure 1G). Many ion channels also carry out functions needed for mechanosensation. Specifically, PIEZO1 can sense mechanical force signals such as fluid shear stress and cell stretching and transduce signals into cells to mediate transcriptional changes (3). Our finding suggested that *Tie1* deletion may alter the way LECs sense mechanical force signals in lymphatics and supported our original hypothesis that ANGPT/TIE signaling must be modulated by mechanical cues. Because *Piezo1*, genes of the ANGPT/TIE pathway, and *Foxo1* have all been shown to modulate lymphatic valve development and function in mice, and mutations in *PIEZO1*, *ANGPT2*, and *TIE1* have been identified in patients with lymphedema, we suspected they might be related in a common network, leading us to explore possible interactions (4, 30, 34).

### PIEZO1 activation regulates FOXO1 localization through ANGPT/TIE signaling.

**Figure 3:**
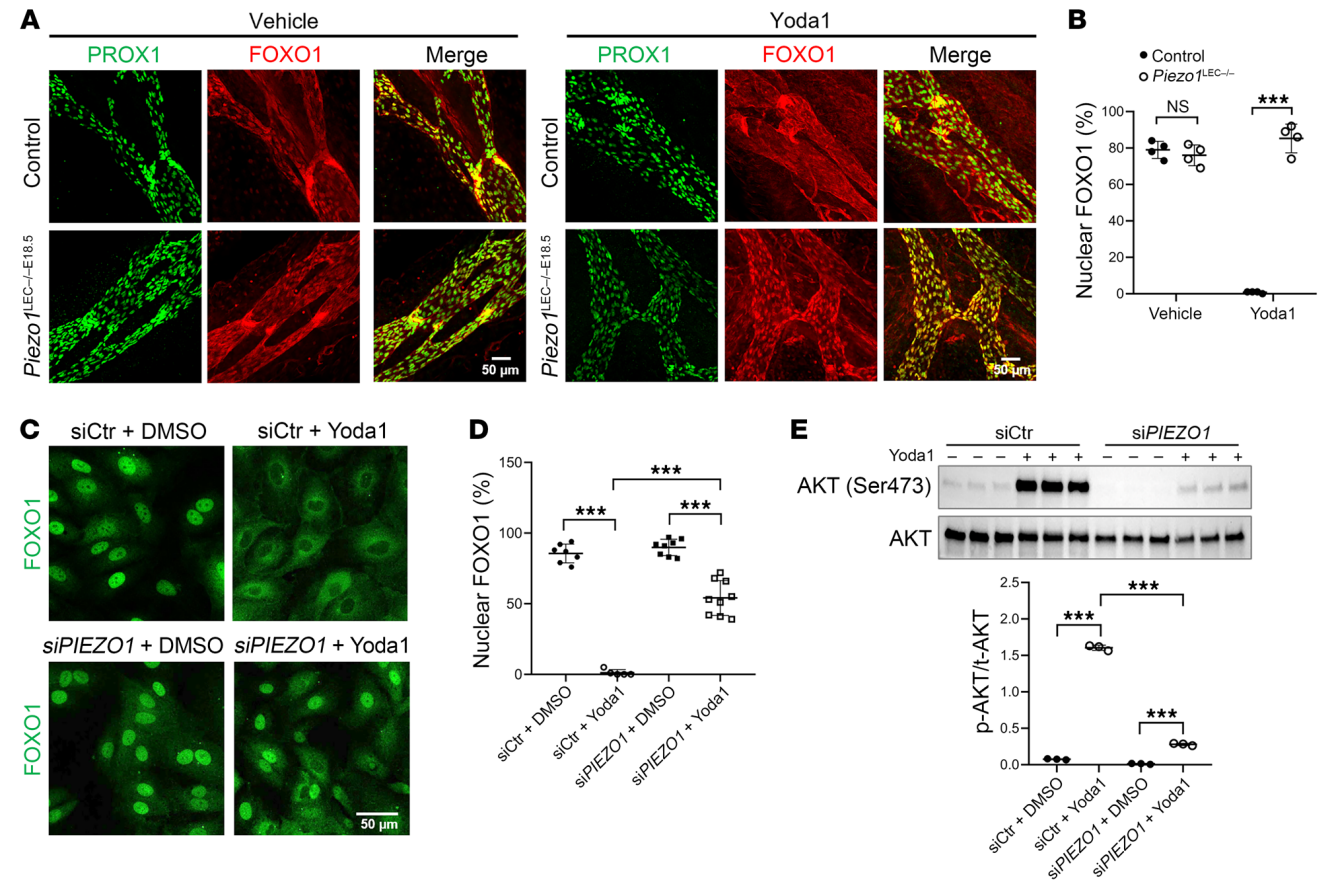
Activation of PIEZO1 signaling promotes nuclear exclusion of FOXO1 and activates the AKT pathway. (**A**) Mesenteries isolated from P1 *Piezo1^WB–/–E18.5^* pups or their littermate controls were subjected to a 30-minute incubation at 37°C with either 250 nM Yoda1 or vehicle. After fixation in 2% PFA for 30 minutes, the mesenteries were stained for PROX1 and FOXO1. Scale bars: 50 μm. (**B**) Quantification of mouse LECs with nuclear FOXO1 localization (*n* = 4 mice in each group). (**C**) HDLECs were transfected with siCtr or si*PIEZO1* for 48 hours and subsequently treated with either vehicle or 250 nM Yoda1 for 30 minutes. Following fixation, cells were stained for FOXO1. Scale bar: 50 μm. (**D**) Quantification of cells exhibiting nuclear FOXO1 staining. (**E**) Western blot analysis of p-AKT levels in HDLECs transfected with siCtr or siPIEZO1 and treated with either vehicle or Yoda1. Each band represents a biological replicate sample (*n* = 3). Data are expressed as the mean ± SD. *P < 0.05, **P < 0.01, and ***P < 0.001, by 2-tailed, unpaired Student’s *t* test (**B**) and 2-way ANOVA followed by Tukey’s multiple-comparison test (**D** and **E**).

Intriguingly, *Piezo1* was altered in LECs from *Tie1*-deficient mice. However, rather than being downregulated like other valve-forming genes (Figure 1G), expression of this gene was upregulated, suggesting it might function upstream of the TIE signaling pathway. Our data, together with prior published work, suggested that PIEZO1, ANGPT/TIE pathways, and FOXO1 might interact in a common molecular pathway to regulate lymphatic development and lymphatic valve formation. To test our hypothesis, we first treated mesenteries isolated from WT and *Piezo1^LEC–/–^* mice ex vivo with vehicle or a small-molecule agonist that activates PIEZO1, Yoda1. Yoda1 rapidly triggered nuclear FOXO1 extrusion in WT mesenteries but not in *Piezo1^LEC–/–^* mesenteries, confirming the PIEZO1/FOXO1 axis in mesenteric lymphatics (Figure 3, A and B). We found that HDLECs treated with Yoda1 also demonstrated nuclear export of FOXO1 (Figure 3, C and D) and AKT phosphorylation (Figure 3E). HDLECs treated with an siRNA against *PIEZO1* largely abolished FOXO1 shuttling (Figure 3, C and D) and AKT activation following Yoda1 treatment (Figure 3E), demonstrating dependence on this channel.

**Figure 4:**
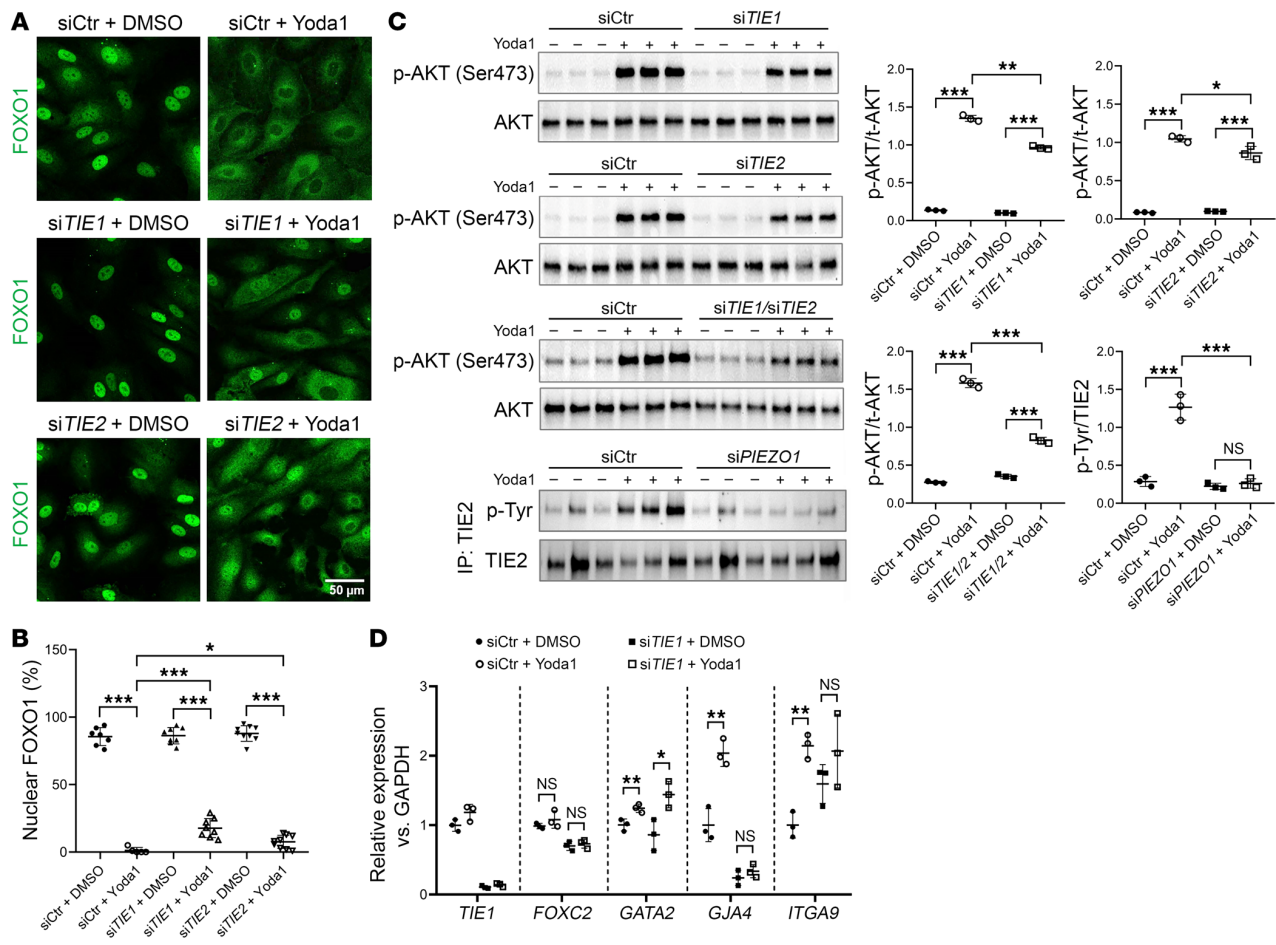
TIE signaling partially mediates AKT/FOXO1 activation triggered by PIEZO1 signaling. (**A**) HDLECs were transfected with siCtr, si*TIE1*, si*TIE2*, or si*TIE1*/si*TIE2* for 48 hours and then exposed to either vehicle or 250 nM Yoda1 for 30 minutes. After fixation, cells were stained for FOXO1. This experiment was carried out concurrently with the one depicted in Figure 3C. The samples used in the siCtr plus DMSO and siCtr plus Yoda1 groups were identical to those in Figure 3C. Scale bar: 50 μm. (**B**) Quantification of cells exhibiting nuclear FOXO1 staining. This experiment was repeated 3 times. (**C**) HDLECs were transfected with the specified siRNAs and treated with vehicle or Yoda1 as described above. Cell lysates were subjected to Western blot analysis to assess AKT phosphorylation. TIE2 was isolated from cell lysates via immunoprecipitation and subsequently analyzed by Western blotting to evaluate its phosphorylation status. Each band represents a biological replicate sample (*n* = 3). (**D**) qPCR analysis of HDLECs transfected with siCtr or si*TIE1* and subsequently treated with Yoda1 (250 nM, 24 hours) or vehicle. Expression levels of *TIE1*, *FOXC2*, *GATA2*, *GJA4*, and *ITGA9* genes were measured. Data are expressed as the mean ± SD. *P < 0.05, **P < 0.01, and ***P < 0.001, by 2-way ANOVA followed by Tukey’s multiple-comparison test (**B** and **C**) and 2-tailed, unpaired Student’s *t* test (**D**).

Next, we wondered if this PIEZO1/FOXO1 effect requires ANGPT/TIE signaling. HDLECs treated with an siRNA against *TIE1* or *TIE2* exhibited increased nuclear retention of FOXO1 (Figure 4, A and B) and decreased AKT phosphorylation in the presence of Yoda1 (Figure 4C). Double-knockdown of both *TIE1* and *TIE2* resulted in a greater decrease of AKT phosphorylation (Figure 4C). Together, these data supported a role for TIE receptors in modulating the effect of PIEZO1 on FOXO1 nuclear localization.

We next sought to determine whether phosphorylation of the TIE receptors occurs following PIEZO1 channel activation and performed Western blot analysis on lysates from HDLECs following immunoprecipitation of the TIE2 receptor. Increased p-TIE2 levels were detected in the Yoda1-treated group, indicating activation of the ANGPT/TIE pathway (Figure 4C) downstream of PIEZO1 activation. Intriguingly, the levels of TIE1 were reduced following PIEZO1 channel activation, which was unexpected and is explained in a later section of this report.

Finally, prior publications have shown that Yoda1 can trigger upregulation of several valve-forming genes in cultured LECs (3). To examine whether this effect is also dependent on TIE1, siCtr- or si*TIE1*-treated HDLECs were treated with DMSO or Yoda1 for 24 hours, and RNA was extracted for qRT-PCR analysis. Expression of *GATA2*, *GJA4*, and *ITGA9*, but not *FOXC2*, was upregulated by Yoda1. *TIE1* knockdown abolished *GJA4* upregulation and mildly affected *ITGA9* (Figure 4D), providing additional support for a shared molecular pathway.

### PIEZO1 activation triggered exocytosis of the ANGPT2 ligand from LECs.

**Figure 5:**
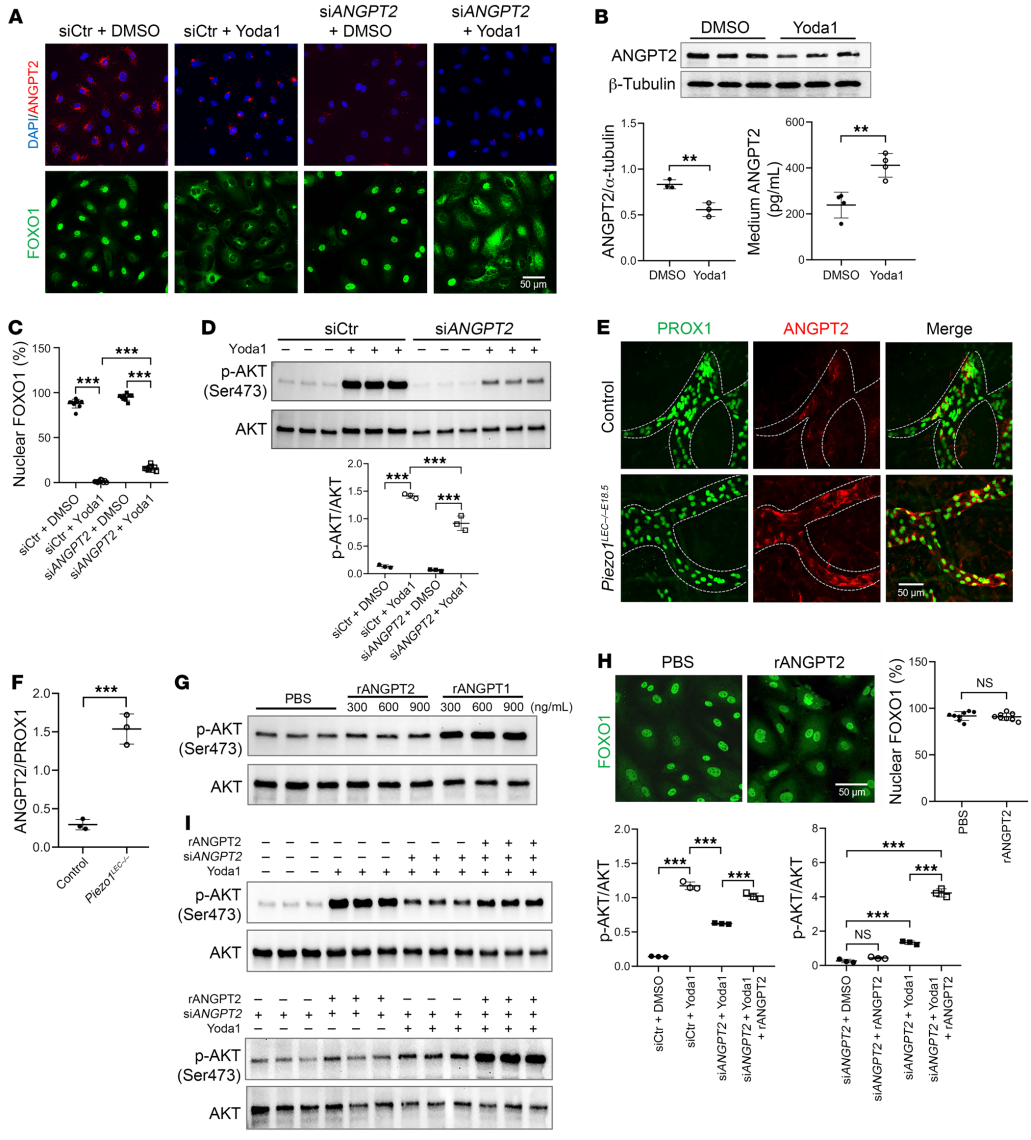
PIEZO1 activation induces ANGPT2 exocytosis in LECs. (**A**) Immunostaining for ANGPT2 and FOXO1 in HDLECs transfected with siCtr or siANGPT2 and treated with DMSO or Yoda1 (250 nM, 30 minutes). Scale bar: 50 μm. (**B**) Western blot analysis of ANGPT2 expression in lysates from HDLECs treated with Yoda1 or DMSO. ANGPT2 concentration in the HDLEC culture medium was measured by ELISA (lower right panel). Each band represents a biological replicate sample (*n* = 3). (**C**) Quantification of cells exhibiting nuclear FOXO1 staining in **A**. This experiment was repeated 3 times, and 3 fields were counted in each group. (**D**) Western blot analysis of p-AKT levels in lysates from HDLECs treated with siCtr or siANGPT2 and DMSO or Yoda1. Each band represents a biological replicate sample (*n* = 3). (**E**) Skins isolated from P1 *Piezo1^WB–/–E18.5^* pups and their littermate controls were stained for PROX1 and ANGPT2. Scale bar: 50 μm. (**F**) Quantification of the ANGPT2^+^ areas in lymphatic vessels from 3 mice in each group. (**G**) Western blot analysis of AKT activation following treatment with rANGPT2 or rANGPT1 at the indicated concentrations for 30 minutes. Each band represents a biological replicate sample (*n* = 3). (**H**) FOXO1 immunostaining of HDLECs treated with vehicle or rANGPT2 (600 ng/mL, 30 minutes) and quantification of cells displaying nuclear FOXO1 staining. Scale bar: 50 μm. (**I**) Western blot analysis of p-AKT levels under the indicated conditions, with rANGPT2 administered at 600 ng/mL and Yoda1 at 250 nM. Each band represents a biological replicate sample (*n* = 3). Data are expressed as the mean ± SD. *P < 0.05, **P < 0.01, and ***P < 0.001, by 2-way ANOVA followed by Tukey’s multiple-comparison test (**C**, **D**, and **I**) and 2-tailed, unpaired Student’s *t* test (**B**, **F**, and **H**).

The observed ability of both Yoda1 and the TIE receptor ligand Hepta-ANG1 to trigger TIE receptor phosphorylation and shuttle FOXO1 and the reduced ability of Yoda1 to cause FOXO1 shuttling in *TIE1*-deficient HDLECs supported our hypothesis that PIEZO1 activation could trigger ANGPT/TIE signaling. Knowing that an influx of intracellular calcium can promote ANGPT2 secretion from LECs and blood ECs (36), we further hypothesized that activation of PIEZO1 by Yoda1 promotes exocytosis of ANGPT2 from HDLECs, resulting in autocrine TIE signaling. Staining of HDLECs with an antibody against ANGPT2 confirmed the presence of intracellular stores of ANGPT2 (Figure 5A). Treatment of HDLECs with Yoda1 resulted in rapid secretion of ANGPT2, demonstrated by decreased intracellular stores (Figure 5A), elevated ANGPT2 levels in the medium as measured by ELISA, and decreased intracellular ANGPT2 protein as determined by Western blotting (Figure 5B). The ability of Yoda1 to shuttle FOXO1 was in part dependent on ANGPT2, as knocking down ANGPT2 using siRNA attenuated FOXO1 nuclear extrusion by Yoda1 treatment (Figure 5, A–C). Yoda1-mediated AKT phosphorylation was also decreased upon ANGPT2 knockdown (Figure 5D).

On the basis of the model that mechanical forces are transient in vivo, we also tested whether FOXO1 shuttling is dynamic and thus reversible. We observed nuclear reaccumulation of FOXO1 60 minutes after Yoda1 removal from the culture medium. By contrast, ANGPT2 intracellular signal did not recover until at least 120 minutes after Yoda1 removal (Supplemental Figure 8).

To provide additional evidence supporting the role of PIEZO1 in the regulation of ANGPT2 exocytosis, we performed immunostaining of ANGPT2 using skin samples from both WT and *Piezo1^LEC–/–^* mice. Remarkably, the deletion of PIEZO1 resulted in the visible accumulation of ANGPT2 within LECs (Figure 5, E and F). This finding further supported a role of PIEZO1 in governing lymphatic ANGPT2 exocytosis.

### ANGPT2 requires ectodomain shedding of TIE1 triggered by PIEZO1 to activate TIE2 and FOXO1 nuclear export.

Despite the clear evidence that PIEZO1 activation triggered exocytosis of ANGPT2, we were surprised that, unlike recombinant ANGPT1 (rANGPT1) treatment, rANGPT2 alone could not activate AKT or trigger FOXO1 nuclear extrusion in HDLECs (Figure 5, G and H). In si*ANGPT2*-treated HDLECs, rANGPT2 could not increase AKT phosphorylation unless combined with Yoda1 treatment (Figure 5I), suggesting that, unlike Hepta-ANG1, ANGPT2 required cofactors or cellular events that were triggered by Yoda1 in order to fully activate TIE/PI3K/AKT in LECs.

**Figure 6:**
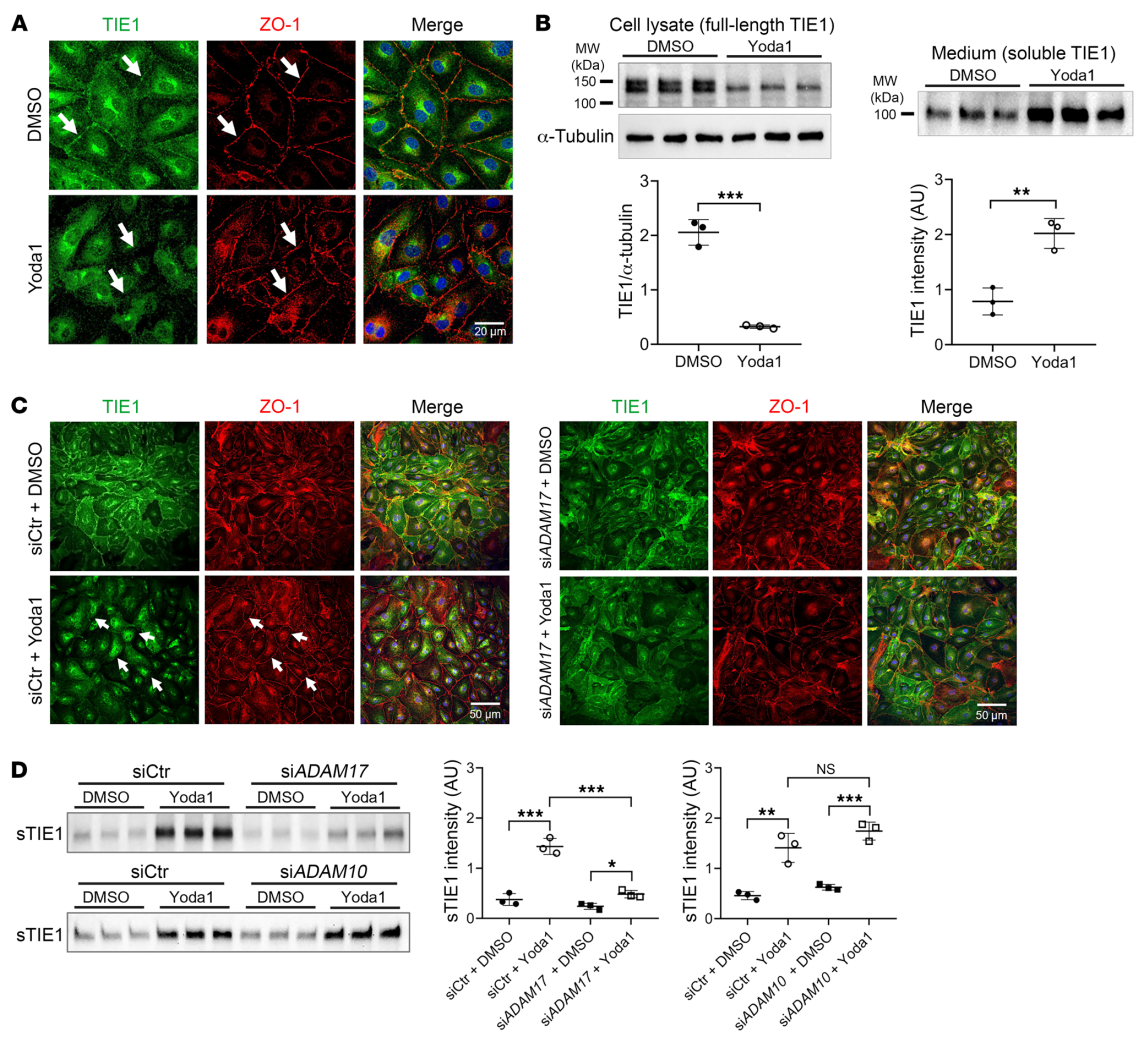
PIEZO1 activation promotes ADAM17-mediated TIE1 shedding in HDLECs. (**A**) Following a 30-minute treatment with Yoda1, HDLECs displayed a reduced distribution of TIE1 at cell-cell junctions (indicated by arrows), as observed in TIE1 and tight-junction protein 1 (ZO-1) immunostaining. Scale bar: 20 μm. (**B**) Yoda1-treated HDLECs exhibited increased TIE1 shedding, as confirmed by Western blot analysis (left panel: cell lysate protein samples; right panel: protein samples obtained from TCA precipitation of the culture medium). Each band represents a biological replicate sample (*n* = 3). (**C**) HDLECs treated with either siCtr or si*ADAM17* were stimulated with Yoda1 or vehicle control, followed by staining for TIE1 and ZO-1. Arrows highlight the areas at cell-cell junctions where TIE1 shedding occurred. Scale bars: 50 μm. (**D**) TIE1 shedding was assessed by Western blot analysis using medium samples subjected to TCA precipitation from siCtr-, si*ADAM17*-, or si*ADAM10*-treated HDLECs after vehicle or Yoda1 treatment. sTIE1, soluble TIE1. Each band represents a biological replicate sample (*n* = 3). Data are expressed as the mean ± SD. *P < 0.05, **P < 0.01, and ***P < 0.001, by 2-tailed, unpaired Student’s *t* test (**B**) or 2-way ANOVA followed by Tukey’s multiple-comparison test (**D**).

When performing Western blot analysis of the TIE1 and TIE2 receptors following PIEZO1 activation in HDLECs, we were also surprised to observe that Yoda1 treatment led to a rapid reduction of TIE1 protein expression on the cell surface. This was confirmed by immunostaining (Figure 6A) and Western blotting (Figure 6B). We then tested media from cells treated with Yoda1 and detected the N-terminus of TIE1, supporting ectodomain shedding of the receptor following activation of PIEZO1 (Figure 6B).

Similar to the dynamic shuttling of FOXO1, the shedding of TIE1 and its subsequent reaccumulation on the cell surface was reversible. When PIEZO1 was no longer activated, TIE1 reaccumulated on the cell membrane, replenishing the cleaved TIE1 within as little as 2 hours (Supplemental Figure 9, A–C).

These data suggested to us that ectodomain shedding of TIE1 was the additional event needed to boost the ability of ANGPT2 to activate the TIE2 receptor. These results are in keeping with prior studies: TIE1 has been reported to be an inhibitory coreceptor of TIE2 (37), and activation of TIE2 by ANGPT2 was prevented by TIE1-TIE2 heterodimerization when tested in TIE-transfected cells (38). In contrast to ANGPT2, ANGPT1 or its mimic Hepta-ANG1 alone was sufficient to activate the ANGPT/TIE pathway (Figure 5G and Supplemental Figure 7), consistent with a context-dependent function of ANGPT2 and prior work showing that ANGPT1 is a more potent agonist than ANGPT2 (18, 39, 40).

### ADAM17 is the sheddase required for TIE1 ectodomain shedding.

Prior reports have demonstrated that TIE1 shedding is an important component in the regulation of vascular homeostasis (37, 41, 42). However, the identity of the sheddase responsible for cleaving TIE1 in lymphatics remained elusive. It has been reported that the activation of PIEZO1 induces the enzymatic activity of the metalloproteinases ADAM metallopeptidase domain 10 (ADAM10) and ADAM17 (43). Here, we pretreated HDLECs with various sheddase inhibitors to investigate their potential in preventing TIE1 shedding following Yoda1 treatment. Notably, the MMP/ADAM17 inhibitor TAPI-2 effectively abolished TIE1 shedding, whereas the ADAM10 inhibitor GI254023X, which also exhibits weak inhibition of ADAM17, modestly attenuated TIE1 shedding (Supplemental Figure 10). These findings suggested that ADAM17 may be responsible for TIE1 cleavage. To validate these findings, we treated HDLECs with siCtr or si*ADAM17* followed by Yoda1 stimulation. Both TIE1 immunostaining and Western blot analyses revealed that ADAM17 knockdown effectively prevented Yoda1-induced TIE1 shedding (Figure 6, C and D), confirming the role of ADAM17 as the sheddase responsible for TIE1 cleavage in LECs. In contrast, pretreatment of HDLECs with an siRNA targeting ADAM10, another principal ADAM sheddase, failed to inhibit Yoda1-induced TIE1 cleavage (Figure 6D).

### Yoda1 induces TIE1 shedding and ANGPT2 exocytosis through calcium influx.

**Figure 7:**
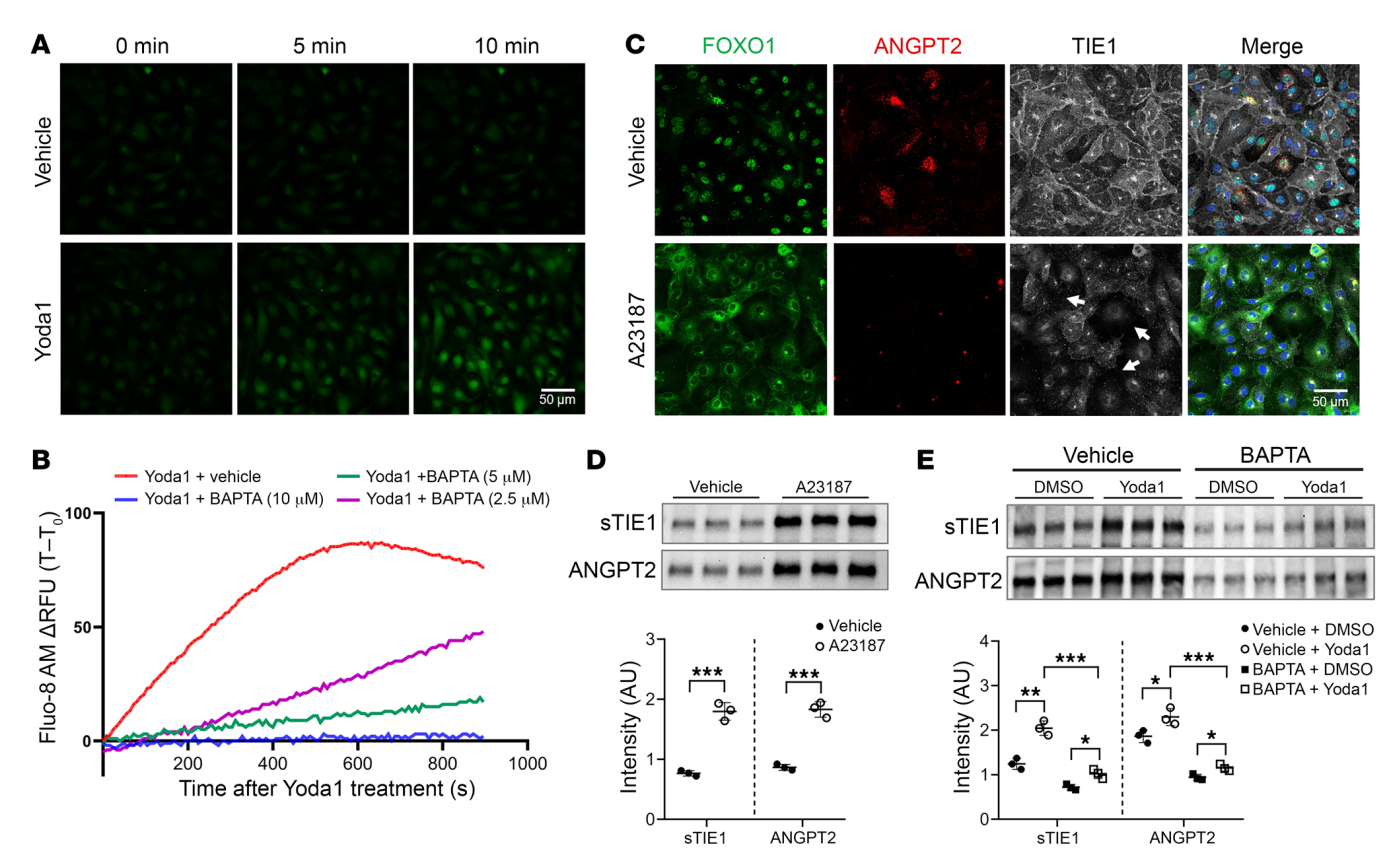
Yoda1 induces TIE1 shedding and ANGPT2 exocytosis via extracellular calcium influx. (**A**) Intracellular calcium levels in HDLECs treated with Yoda1 or vehicle were visualized using confocal microscopy with the cell-permeable Ca^2+^ indicator Fluo-8 AM. Scale bar: 50 μm. (**B**) Quantification of Yoda1-induced calcium influx in HDLECs pretreated with varying concentrations of the calcium chelator BAPTA. RFU, relative fluorescence units; T–T_0_, difference between the value measured at time point (T) and the value measured immediately prior to the treatment (T_0_). (**C**) Immunostaining for FOXO1, ANGPT2, and TIE1 in HDLECs treated with either vehicle or the calcium ionophore A23187 for 30 minutes. Arrows indicate the areas at cell-cell junctions where TIE1 shedding occurred. Scale bar: 50 μm. (**D**) Western blot analysis of TIE1 shedding and ANGPT2 exocytosis in HDLECs treated with vehicle or A23187. Each band represents a biological replicate sample (*n* = 3). (**E**) Western blot analysis of Yoda1-triggered TIE1 shedding and ANGPT2 exocytosis in the presence or absence of BAPTA. Each band represents a biological replicate sample (*n* = 3). Data are expressed as the mean ± SD. **P < 0.01 and ***P < 0.001, by 2-tailed, unpaired Student’s *t* test (**D**) and 2-way ANOVA followed by Tukey’s multiple-comparison test (**E**).

PIEZO1 mediates the transduction of extracellular mechanical forces into cellular responses by facilitating the entry of cations, including calcium and sodium, thereby initiating a cascade of signaling pathways (44, 45). To determine whether Yoda1 induces TIE1 shedding and ANGPT2 exocytosis through calcium influx, we monitored intracellular calcium levels using the fluorescent calcium indicator Fluo-8 AM. Treatment with Yoda1 produced a gradual increase in intracellular calcium levels, consistent with activation of the PIEZO1 channel (Figure 7, A and B). We also exposed HDLECs to the calcium ionophore A23187, a potent inducer of rapid calcium influx. Similar to Yoda1, A23187 robustly triggered FOXO1 translocation, ANGPT2 exocytosis, and TIE1 shedding in HDLECs (Figure 7, C and D), suggesting that these cellular responses can indeed be triggered by elevated intracellular calcium concentrations. Furthermore, we used the cell-permeable calcium chelator BAPTA to selectively deplete intracellular calcium; this intervention significantly attenuated the capacity of Yoda1 to induce TIE1 shedding and ANGPT2 exocytosis (Figure 7E), supporting a key role of intracellular calcium in mediating these PIEZO1-associated cellular responses.

## Discussion

A functional lymphatic system is needed for tissue homeostasis, and defects in lymphatic function and growth underlie lymphedema and contribute to many disease states (reviewed in ref. 46). In recent years, a number of genes and pathways have been identified that are essential for lymphatic development and function, including the ANGPT2 vascular growth factor and its receptors TIE1/TIE2, as well as environmental cues such as mechanical forces (15–18, 24, 25, 47). Here, we identified a molecular network linking activation of the mechanosensory cation channel PIEZO1 to the FOXO1 transcriptional program in LECs that acts, in part, through the ANGPT/TIE pathway. Our data support a model in which PIEZO1 activation leads to rapid secretion of ANGPT2 ligand and ectodomain shedding of TIE1. We found that delivery of secreted ANGPT2 ligand to the surface of LECs activated the TIE2 receptor in an autocrine fashion, triggering nuclear extrusion of the FOXO1 gene downstream of PI3K/AKT activation. Subsequent reduction in the nuclear activity of FOXO1 led to decreased expression of the transcriptional repressor *Prdm1*, relieving the repression of key lymphatic genes such as *Gja4* (Cx37) and *Foxc2* and prompting cell behavior changes needed for lymphatic valve leaflet formation. This molecular pathway ensures that lymphatic endothelial cellular responses to mechanical cues and/or other external stimuli that trigger calcium influx into the cell occur in a highly orchestrated, transient, and localized fashion needed for the formation of a functional lymphatic system.

Importantly, studies have demonstrated key roles for all the genes in this molecular pathway for lymphatic development and function — in humans, mice, or both. Mutations in *ANGPT2* and *PIEZO1* and variants in the gene encoding the *TIE1* receptor have been associated with lymphedema in patients, and loss-of-function mutations in *Angpt2*, *Tie1*, *Tie2*, *Piezo1*, *Foxc2*, and *Gja4* lead to lymphatic defects in mice including failure of lymphatic valve formation, a process dictated by local OSS (3, 5–9, 18, 24, 25, 48–50). By contrast, postnatal loss of FOXO1 leads to ectopic formation of lymphatic valves in mice, although earlier deletion of FOXO1 in mice leads to lymphatic vessel defects, underscoring the critical importance of tight and dynamic regulation of FOXO1 levels (34, 51). Our model demonstrates how transient downregulation of FOXO1, which must happen to allow valve formation, can be accomplished through PIEZO1 and ANGPT/TIE signaling. Anatomically, lymphatic valves form in predictable locations, at sites of OSS, but until now, how all these genes might interact in a pathway or how mechanical cues are sensed by LECs to elicit a response has not been shown.

The key data that led to the discovery of this network came from our analysis of RNA-Seq data isolated from lymphatics of *Tie1*-deficient embryos; as expected, several genes expressed in LECs were downregulated, but it was also evident that FOXO1 downstream transcriptional targets were increased. Transcriptional activity of FOXO1 can be rapidly regulated by its phosphorylation and subsequent extrusion/shuttling from the nucleus. In keeping with the RNA-Seq results, the potent TIE2 activator Hepta-ANG1 led to rapid nuclear extrusion of FOXO1 from LECs in vitro and in vivo, and we confirmed that this shuttling was reduced in *TIE1*-deficient human cells in culture and mesenteric lymphatics in *Tie1-*deficient mice in vivo.

Although we believe the FOXO1 results were significant and helped to explain many of the observed phenotypes, most excitingly and unexpectedly, we observed increased expression of genes encoding numerous ion channels, including the nonselective cation channel PIEZO1. *Piezo1* was of interest for several reasons: (a) it is activated by mechanical forces including OSS, and (b) mutations in this gene have been identified in patients with lymphedema, and mice lacking PIEZO1 develop lymphatic defects, including failure of valve formation. Intriguingly, expression of this gene was increased rather than decreased in our data set, suggesting it might function upstream of TIE1. In keeping with this model, activation of PIEZO1 with the small molecule Yoda1 led to cellular events in LECs similar to those seen following treatment with Hepta-ANG1, including AKT activation and nuclear exclusion of FOXO1. Yoda1 treatment also led to rapid exocytosis of ANGPT2, whereas knockdown of *ANGPT2* in HDLECs using the siRNA blunted the effect of Yoda1 on FOXO1 export, providing a model to explain how PIEZO1 activation promotes TIE signaling in the lymphatics in vivo.

It is notable that this system and pathway should allow exquisite regulation and response of LECs to local and transient forces. Furthermore, release of ANGPT2 will deplete the cell of ligand and ultimately lead to the termination of receptor activation, permitting reaccumulation of nuclear FOXO1 and transcriptional upregulation of *ANGPT2* to eventually replete the vesicular stores. Our data confirmed reaccumulation of FOXO1 in the nucleus of HDLECs within 60 minutes after removal of the channel activator, Yoda1.

Intriguingly, recombinant ANGPT2 was unable to trigger AKT phosphorylation, but combined treatment with Yoda1 and rANGPT2 could rescue AKT phosphorylation in si*ANGPT2*-treated HDLECs, suggesting the need for a cofactor or an additional event for a full cellular response triggered by Yoda1. Previously, it was shown that autocrine ANGPT2 signaling can activate TIE2, whereas non-cell-autonomous signaling has a less potent effect (52, 53); various proposed explanations focused on the level of expression and the location of expression of the ANGPT2 ligand but cannot explain the inability of recombinant protein to initiate a response. Our data suggest that ectodomain shedding of TIE1 is needed permissively to permit ANGPT2 to function as a TIE2 agonist, consistent with other models demonstrating that reducing TIE1 enhances TIE2 activation by ANGPT2 but not ANGPT1 (38).

The requirement for TIE1 cleavage for TIE2 activation by ANGPT2 provides an additional level to fine-tune molecular events. TIE1 heterocomplex formation with TIE2 reduces autophosphorylation of the Y1102 tyrosine residue of TIE2, limiting the downstream PI3K/AKT signaling that triggers FOXO1 nuclear shuttling (38). While downregulation of FOXO1 activity is needed for lymphatic valve formation, at other times during lymphatic vessel development, FOXO1 activity is required (18). As a transcription factor, FOXO1 is active when in the nucleus and inactive when shuttled to the cytoplasm. In lymphatic development, embryonic deletion of TIE1 led to a widespread reduction of TIE2 on the cell surface and resulted in the accumulation of ANGPT2 in LECs (Supplemental Figure 5B and Supplemental Figure 6A). In the early postnatal period, TIE1 and TIE2 exhibited comparable distribution patterns in lymphatics, with the exception of valve regions where TIE1 was more highly expressed together with ANGPT2 and where FOXO1 shuttling occurred (Supplemental Figures 5 and 6). The presence of TIE1 may help to control precocious FOXO1 shuttling by modulating the phosphorylation status of TIE2 until the appropriate OSS or extracellular signal is sensed and ANGPT2 is released. Finally, metalloproteases have been implicated in ectodomain shedding of TIE1 (54), and HDLECs and LECs in vivo express several metalloproteases including MMP2, MMP9, and ADAM17. Indeed, Yoda1 has been shown to activate metalloproteases (43, 55), providing a biologically plausible explanation. We confirmed that the sheddase involved in Yoda1-triggered TIE1 shedding in LECs was ADAM17.

**Figure 8:**
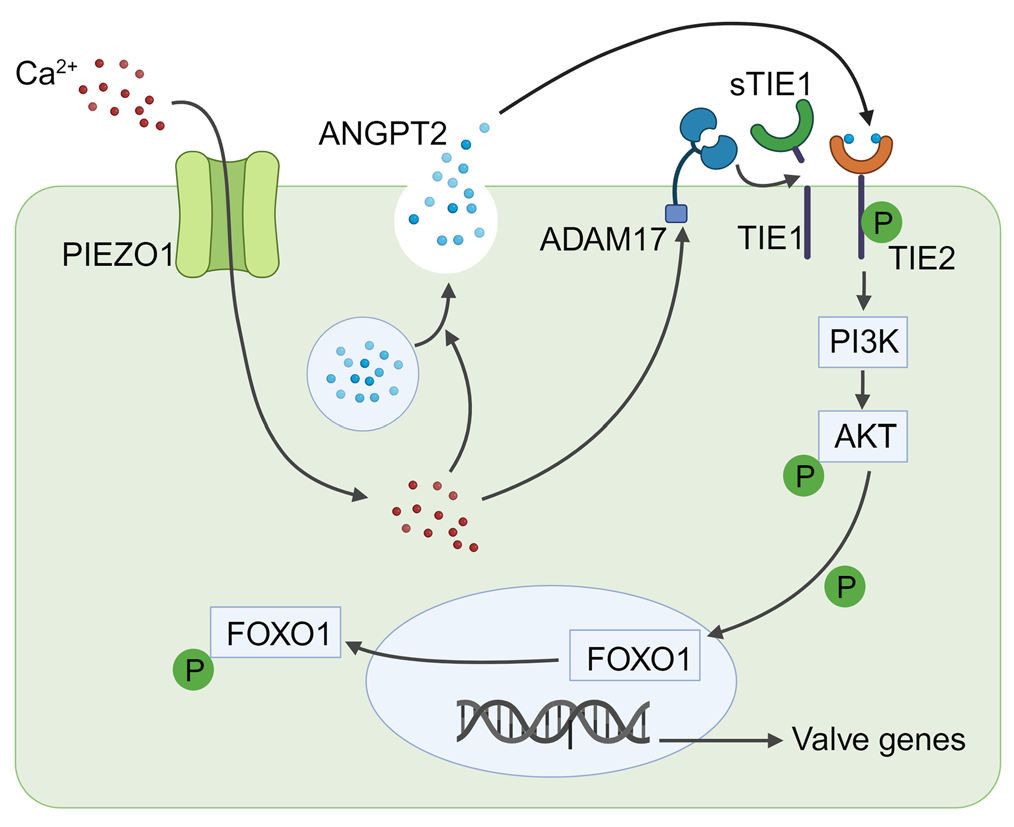
Model of the PIEZO1/ANGPT/TIE/FOXO1 axis in the regulation of lymphatic development. Activation of the mechanosensory cation channel PIEZO1 initiates a cascade of events crucial for lymphatic development. This activation leads to an increase in intracellular calcium levels, subsequently triggering the release of ANGPT2 from intracellular vesicles and activation of the protease ADAM17. ADAM17 cleaves cell membrane–anchored TIE1, facilitating the binding and activation of TIE2 by the released ANGPT2. This activation, in turn, initiates downstream signaling through the PI3K/AKT/FOXO1 pathways. The translocation of FOXO1 from the nucleus to the cytoplasm alleviates its repression of lymphatic valve– and other lymphatic-associated genes that are crucial for lymphatic development. This finely orchestrated axis plays a pivotal role in governing the intricate processes involved in the formation and maturation of the lymphatic system.

Taken together, our results demonstrate a molecular pathway from an extrinsic cue to a membrane signaling cascade and intracellular events critical for many aspects of lymphatic remodeling and morphology (Figure 8). Importantly, these genes all play important roles in lymphatic development and function in mice and humans. Future work will help to determine how these pathways might be modulated for therapeutic benefit in lymphatic-associated diseases and tissue engineering.

### Limitations.

PIEZO1 is a nonspecific cation channel that can be activated by several extrinsic stimuli including OSS, cellular stretch, extracellular matrix stiffness, membrane curvature, etc. Although OSS has been shown to trigger the lymphatic valve–forming gene process, it is unclear whether the force needed to activate PIEZO1 occurs in lymphatic vessels in vivo. In one study, the instantaneous activation threshold for PIEZO1 was demonstrated to be approximately 57 dynes/cm^2^ (45), which is much higher than is predicted to occur in lymphatic vessels. The physiologic signal for PIEZO1 activation to trigger ANGPT/TIE signaling and FOXO1 translocation is currently unknown and could not be adequately addressed with the currently available experimental models. Future research using compound, genetically engineered animal models is needed to further validate the proposed mechanism in vivo and to investigate the intricate interactions within these signaling pathways. Furthermore, whether compound genetic variants in genes in the PIEZO1/ANGPT2/TIE/FOXO1 pathway predispose to lymphatic disorders in humans was not explored here and remains an interesting path for future investigation.

## Methods

### Sex as a biological variable.

In this study, animals of both male and female sexes were used, and sex was not considered a biological variable.

### Mice and breeding.

The following mouse lines were used for our study: *Tie1^fl/fl^* (22), *Piezo1^fl/fl^* *Prox1*-CreER^T2^ (a gift from Young-Kwon Hong, University of Southern California, Los Angeles, California, USA) (3), and *Pdpn*Cre (a gift from Guillermo Oliver, Northwestern University, Chicago, Illinois, USA) (29). To generate *Tie1^fl/fl^* mice, a floxed conditional allele of *Tie1* was created using a targeted ES clone (EPD0735_3_C06), whereby tandem bacterial lacZ (β-galactosidase) and neomycin resistance (neor) selection cassettes were incorporated between exons 7 and 8 of the mouse *Tie1* locus, with additional loxP (Cre recombinase recognition) sites flanking exons 8 and 9, obtained from the Knockout Mouse Project (KOMP) repository (https://www.komp.org). Correctly targeted ES clones were used for blastocyst injection and generation of mouse chimera. Subsequent offspring mice harboring the targeted allele (*Tie1^neo^*) were evaluated for gross phenotype. A conditional floxed *Tie1* allele (*Tie1^fl^*) was generated by breeding *Tie1^neo^* mice with Flp recombinase–transgenic mice, resulting in the removal of the neor expression cassettes. Whole-body (*Tie1^WB–/–^*) and lymphatic endothelium–targeted (*Tie1^LEC–/–^*) mice were derived from crosses between *Tie1^fl^* mice with the driver strains RosartTA:tetOCre and *Pdpn*Cre, respectively. Whole-body timed deletion of *Tie1* was achieved by giving 0.5% (wt/vol) Dox-containing drinking water (contain 5% sucrose) to the *Tie1*RosartTA: tetOCre mice for the indicated time periods.

For embryo experiments, breeding pairs were set up in the afternoon, and plugs were verified in the morning. Embryonic age was determined according to the day of the vaginal plug (E0.5).

### Primers and antibodies.

The sequences of the primers used are listed in Supplemental Table 1. Details on the antibodies used are provided in Supplemental Table 2.

### Fluorescence microscopy.

For skin whole-mount preparations, whole embryos up to E16.5 were fixed in 2% PFA overnight and then washed twice with PBS. For embryos of E17.5 and older, skin around the lower belly to the hind limb was separated before fixing with paraformaldehyde (PFA). To evaluate lymphangiogenesis and general lymphatic vessel morphology, dorsal skin was cut from the armpit to the hind limb and then fixed with PFA. Skin patches were permeabilized and blocked in permeabilization/blocking (perm/block) buffer (PBS containing 0.2% Triton X-100, 3% donkey serum, and 3% BSA) for 1 hour. Depending on the embryonic stage, skin patches were incubated with primary antibodies for 1–2 days (1 for E16.5 and below, 2 for E17.5 and above), followed by secondary antibodies overnight at 4°C. Flat-mount of skin patches were imaged with a Nikon A1 confocal microscope. For the back skin lymphangiogenic and mesentery studies, multiple images were taken and stitched together using Nikon NIS Elements software.

For embryonic lymph sac analysis, E15.5 embryos were fixed in 4% PFA overnight and embedded in paraffin for frontal sections. Sections of the lymph sac area were collected and underwent dewaxing and rehydration. Antigen retrieval was performed to enhance staining with citrate buffer in a pressure cooker for 30 minutes. Sections were blocked and permeabilized, followed by staining with the indicated antibodies.

For mesentery whole-mount preparation, mesenteries were isolated from the mice and fixed in 2% PFA for 1 hour. In some experiments, mesenteries were treated with PBS or Yoda1 for 30 minutes at 37°C before fixation. After fixation, mesenteries were washed twice with PBS, permeabilized and blocked in perm/block buffer for 1 hour, and incubated with primary antibodies overnight at 4°C. After 3 washes with PBS, mesenteries were labeled with secondary antibodies overnight at 4°C and imaged with a Nikon A1 confocal microscope.

For cell samples, cells cultured in gelatin-coated (MilliporeSigma, G1393), glass-bottomed plates were fixed in 4% PFA for 10 minutes, permeabilized in PBS containing 0.1% Triton X-100 for 10 minutes, and blocked with PBS containing 5% donkey serum for 1 hour at room temperature, followed by staining with antibodies that were diluted in blocking buffer. Fluorescence images were taken with a Nikon A1 confocal microscope. All images were analyzed with Fiji software.

### Dermal LEC collection and RNA-Seq.

Skin samples were collected from E18.5 embryos (*n* = 2 for each group) and cut into small pieces in RPMI 1640 medium in a 2 mL Eppendorf tube. Each skin sample was digested in the enzyme cocktail that contained dispase II (MilliporeSigma, D4693, 0.4 mg/mL), collagenase type 2 (Worthington Biochemicals, LS004716, 2.5 mg/mL), and DNase I (MilliporeSigma, DN25, 0.1 mg/mL) at 37°C for 1 hour with rotation. In the middle of the digestion, skin pieces were passed through a 1 mm pipette tip 3–5 times to further dissociate the skin. After incubation, a syringe plunger and 100 μm cell strainer were used to rub and filter the digested tissue into a 50 mL flacon tube. The strainer was thoroughly rinsed with cold PBS to recover the digested cells. The 50 mL tubes were spun at 300*g* for 10 minutes. Cell pellets were collected by centrifuge at 300*g* for 10 minutes, and RBCs were lysed using RBC Lysis Buffer (Cell Signaling Technology, 46232). Cells were washed with PBS, stained with fixable viability dye, and labeled with fluorochrome-labeled antibodies against CD45, CD31, and LYVE1 in FACS buffer. Live single cells that were CD45^–^ and CD31^+^ were defined as ECs. Sorting was performed on a FACS Aria III flow cytometer with FACSDiva software (BD Biosciences) using a 100 μm nozzle. LYVE1^hi^ ECs were sorted directly into lysis buffer for RNA extraction using the RNeasy Plus Micro kit (QIAGEN, 74034). The quality of isolated RNA was checked using the Agilent 2100 Bioanalyzer. Library preparation was based on SMART-Seq v4 chemistry, which uses oligo(dT) to prime for full-length cDNA generation. The libraries were sequenced on the HiSeq 4000 system (1 × 50 bp reads, 20–25 million reads per sample). RNA-Seq reads were mapped to the mouse genome reference (mm10) using TopHat. Differential gene expression analysis was performed using the edgeR package in R. Gene expression changes were visualized in heatmaps using the ggplot2 package in R. Volcano plots of differentially expressed genes were generated using EnhancedVolcano R package. Genes with an adjusted *P* value of less than 0.05 and a log fold change of greater than 0.6 were labeled as significantly differentially expressed. Overlap analysis was done using the GeneVenn online program (http://genevenn.sourceforge.net). Venn diagrams were generated using Venn Diagram Maker Online (https://www.meta-chart.com/venn).

### Cell culture.

Primary HDLECs (PromoCell, C-12216) were maintained in EBM-2 MV media (PromoCell, C-22121) on gelatin-coated cell culture plates at 37°C with 5% CO_2_. Cells at passages between 3 and 6 were used for experiments.

### siRNA transfection.

Silencer Select negative siCtr (s4390843) and siRNA targeting *TIE1* (s14141), *TIE2* (s13983), *ANGPT2* (s1359), *ADAM10* (s1004), and *ADAM17* (s13719) were purchased from Thermo Fisher Scientific. siRNAs targeting *FKHR/FOXO1* (sc-35382) and *PIEZO1* (sc-93227) were purchased from Santa Cruz Biotechnology. To knock down each gene, subconfluent cells were transfected with the siRNA using RNAimax reagent (Invitrogen, Thermo Fisher Scientific, 13778075) according to the manufacturer’s instruction. Forty-eight hours after transfection, cells were serum starved in Endothelial Cell Basal Medium MV2 (PromoCell, c-22221) for 4 hours and then stimulated with vehicle, ANGPT1 (R&D System, 923-AN), Hepta-ANG1, rANGPT2 (R&D Systems, 623-AN), or Yoda1 (R&D Systems, 5586).

### Intracellular calcium measurement.

HDLECs grown on gelatin-coated, glass-bottomed dishes (for fluorescence imaging) or 96-well, white cell culture plates (for fluorescence intensity reading) were serum starved for 4 hours and then loaded with 4 μM Fluo-8 AM (AAT Bioquest, 21082) for 30 minutes at 37°C. Cells were washed twice with warm Endothelial Cell Basal Medium MV2. For fluorescence imaging, vehicle or Yoda1 (250 nM) was directly added to the dishes, and the cells were imaged using a Nikon A1 confocal microscope. For fluorescence quantification, cells were pretreated with different doses of the calcium chelator 1,2-bis (*o*-aminophenoxy) ethane-N, N, N′, N′-tetra-acetic acid (BAPTA) for 10 minutes, followed by vehicle or Yoda1 (250 nM) treatment. The fluorescence intensity was measured continuously using the BioTek Synergy HT Microplate Reader.

### Biotinylation and immunoprecipitation of cell-surface proteins.

HDLECs were washed 3 times with ice-cold PBS, followed by a 30-minute incubation at 4°C in a 0.5 mg/mL solution of sulfo-NHS-SS-Biotin (Thermo Fisher Scientific, A39258) prepared in PBS supplemented with CaCl_2_ and MgCl_2_. To quench the biotinylation reaction, cells were washed 3 times with 50 mM glycine dissolved in PBS containing CaCl_2_ and MgCl_2_, with each wash including a 5-minute incubation. Subsequently, cells were lysed using a Triton X-100 lysis buffer supplemented with a protease inhibitor cocktail (MilliporeSigma, P8340), and the lysate was then centrifuged at 14,000*g* for 20 minutes to obtain a clear supernatant. Biotinylated cell-surface proteins were then selectively immunoprecipitated using avidin agarose resin (Thermo Fisher Scientific, 20219) according to the manufacturer’s instructions.

### ANGPT2 ELISA.

HDLECs were cultured in gelatin-coated, 12-well plates until nearly confluent. Cells were serum starved for 4 hours in 0.5 mL prewarmed Endothelial Cell Basal Medium MV2 before Yoda1 or DMSO was added. After incubation for 30 minutes, supernatant was collected and centrifuged at 300*g* for 10 minutes to remove cell debris. ANGPT2 ELISA was carried out using the human ANGPT2 Quantikine ELISA Kit (R&D Systems, DANG20) according to the manufacturer’s instructions.

### qRT-PCR.

Total RNA from cells or tissue was isolated using the RNeasy Plus Micro kit (QIAGEN, 74034) or the RNeasy Plus Mini Kit (QIAGEN, 74104). Quality control of samples was carried out using the Eppendorf Biospectrometer. RNA was reverse transcribed using the iScript cDNA Synthesis Kit (Bio-Rad, 1708891). qRT-PCR reactions were carried out in triplicate using the iTaq Universal SYBR Green Supermix (Bio-Rad, 1725124) on a QuantStudio 3 cycler (Thermo Fisher Scientific) according to a standardized protocol.

### Preparation of protein samples and Western blotting.

Cells in each well of a 6-well plate were lysed with 100 μL Laemmli Sample Buffer for direct Western blotting or with RIPA buffer supplemented with a proteinase inhibitor cocktail (MilliporeSigma, P8340) and a phosphatase inhibitor cocktail (MilliporeSigma, P5726) for immunoprecipitation. For immunoprecipitation of TIE1 or TIE2, cell lysate was incubated with 1 μg goat anti-TIE1 antibody (R&D Systems, AF619) or goat anti-TIE2 antibody (R&D Systems, AF313) for 2 hours with rocking at 4°C. After incubation, 25 μL prewashed Protein G Mag Sepharose (Cytiva, 28951379) was added and incubated at 4°C overnight with rocking. On the second day, beads were collected using a magnetic tube rack and washed 3 times with washing buffer (TBS containing 1% Triton X-100, a phosphatase inhibitor cocktail, and a protease inhibitor cocktail, pH7.4). Bound proteins were eluted by removing all wash buffer and adding 50 μL ×2 Laemmli sample buffer containing 50 μM Tris(2-carboxyethyl)phosphine hydrochloride (TCEP), followed by boiling for 3 minutes. For precipitation of total proteins from cell culture medium, 100% trichloroacetic acid (TCA) was added to the medium to a final concentration of 20%. After incubating overnight at 4°C, the pellets were collected by centrifugation (18,000*g* for 30 minutes) and then washed twice with cold acetone. The protein pellets were dried and dissolved in ×2 Laemmli sample buffer containing 50 μM TCEP. Total cell lysates, immunoprecipitated proteins, or TCA precipitated proteins were separated in SDS-PAGE and then transferred onto a TransBlot Turbo Mini-size PVDF membrane (Bio-Rad Laboratories) using the Trans-Blot Turbo Transfer System (Bio-Rad Laboratories). Membranes were blocked with 5% BSA in TBST before being probed first with primary antibodies and then with HRP-conjugated secondary antibodies (Jackson ImmunoResearch), followed by ECL detection reagent (Thermo Fisher Scientific). Western blot images were captured using the iBright Imaging system (Thermo Fisher Scientific).

### Statistics.

Analysis of data was performed using GraphPad Prism 5 (GraphPad Software), Quantification data are expressed as the mean ± SD. Reported *P* values were obtained using 2-tailed unpaired Student’s *t* test or 2-way ANOVA followed by Tukey’s multiple-comparison test as appropriate. A *P* value of less than 0.05 was considered statistically significant.

### Study approval.

All animal experiments were approved by the IACUC of the Center for Comparative Medicine at Northwestern University.

### Data availability.

RNA-Seq data in this study have been deposited in the NCBI’s Gene Expression Omnibus (GEO) database (GEO GSE211849). Values for all data points in graphs are available in the Supporting Data Values file.

## Author contributions

SEQ, JD, P Liu, JJ, and BRT designed the research studies. SEQ, JD, and P Liu wrote the manuscript, and SEQ, JD, P Liu, JD, BRT, YZ edited the manuscript. SEQ, JD, P Liu, JJ, BT, and YZ analyzed data. JD, P Liu, IS, P Leeaw, and SM conducted experiments. JD initiated the study, and JD and P Liu provided equal contributions to data acquisition and interpretation. The order of the co–first authors’ names was determined on the basis of their contributions to this study.

## Supplementary Material

Supplemental data

Supporting data values

## Version 1 05/15/2024

Electronic publication

## References

[1] Schwartz MA, Simons M (2012). Lymphatics thrive on stress: mechanical force in lymphatic development. EMBO J.

[2] Choi D (2022). Piezo1-regulated mechanotransduction controls flow-activated lymphatic expansion. Circ Res.

[3] Choi D (2019). Piezo1 incorporates mechanical force signals into the genetic program that governs lymphatic valve development and maintenance. JCI Insight.

[4] Nonomura K (2018). Mechanically activated ion channel PIEZO1 is required for lymphatic valve formation. Proc Natl Acad Sci U S A.

[5] Han S (2022). A novel homozygous missense mutation of *PIEZO1* leading to lymphatic malformation-6 identified in a family with three adverse pregnancy outcomes due to nonimmune fetal hydrops. Front Genet.

[6] Fotiou E (2015). Novel mutations in PIEZO1 cause an autosomal recessive generalized lymphatic dysplasia with non-immune hydrops fetalis. Nat Commun.

[7] Lukacs V (2015). Impaired PIEZO1 function in patients with a novel autosomal recessive congenital lymphatic dysplasia. Nat Commun.

[8] Sabine A (2015). FOXC2 and fluid shear stress stabilize postnatal lymphatic vasculature. J Clin Invest.

[9] Norden PR (2020). Shear stimulation of FOXC1 and FOXC2 differentially regulates cytoskeletal activity during lymphatic valve maturation. Elife.

[10] Karkkainen MJ (2004). Vascular endothelial growth factor C is required for sprouting of the first lymphatic vessels from embryonic veins. Nat Immunol.

[11] Zhang Y (2018). Heterogeneity in VEGFR3 levels drives lymphatic vessel hyperplasia through cell-autonomous and non-cell-autonomous mechanisms. Nat Commun.

[12] Ghalamkarpour A (2009). Recessive primary congenital lymphoedema caused by a VEGFR3 mutation. J Med Genet.

[13] Irrthum A (2000). Congenital hereditary lymphedema caused by a mutation that inactivates VEGFR3 tyrosine kinase. Am J Hum Genet.

[14] D’Amico G (2010). Loss of endothelial Tie1 receptor impairs lymphatic vessel development-brief report. Arterioscler Thromb Vasc Biol.

[15] Qu X (2010). Abnormal embryonic lymphatic vessel development in Tie1 hypomorphic mice. Development.

[16] Partanen J, Dumont DJ (1999). Functions of Tie1 and Tie2 receptor tyrosine kinases in vascular development. Curr Top Microbiol Immunol.

[17] Gale NW (2002). Angiopoietin-2 is required for postnatal angiogenesis and lymphatic patterning, and only the latter role is rescued by angiopoietin-1. Dev Cell.

[18] Souma T (2018). Context-dependent functions of angiopoietin 2 are determined by the endothelial phosphatase VEPTP. Proc Natl Acad Sci U S A.

[19] Leppanen VM (2017). Structural basis of Tie2 activation and Tie2/Tie1 heterodimerization. Proc Natl Acad Sci U S A.

[20] Shen B (2014). Genetic dissection of tie pathway in mouse lymphatic maturation and valve development. Arterioscler Thromb Vasc Biol.

[21] Suri C (1996). Requisite role of angiopoietin-1, a ligand for the TIE2 receptor, during embryonic angiogenesis. Cell.

[22] Jeansson M (2011). Angiopoietin-1 is essential in mouse vasculature during development and in response to injury. J Clin Invest.

[23] Korhonen EA (2022). Lymphangiogenesis requires Ang2/Tie/PI3K signaling for VEGFR3 cell-surface expression. J Clin Invest.

[24] Leppanen VM (2020). Characterization of *ANGPT2* mutations associated with primary lymphedema. Sci Transl Med.

[25] Michelini S (2020). *TIE1* as a candidate gene for lymphatic malformations with or without lymphedema. Int J Mol Sci.

[27] Woo KV, Baldwin HS (2011). Role of Tie1 in shear stress and atherosclerosis. Trends Cardiovasc Med.

[28] Qu X (2019). Loss of flow responsive Tie1 results in impairedaortic valve remodeling. Dev Biol.

[29] Gil HJ (2018). A novel podoplanin-GFPCre mouse strain for gene deletion in lymphatic endothelial cells. Genesis.

[30] Niimi K (2021). FOXO1 represses lymphatic valve formation and maintenance via PRDM1. Cell Rep.

[31] Andrade J (2021). Control of endothelial quiescence by FOXO-regulated metabolites. Nat Cell Biol.

[32] Fukumoto M (2018). Tip-cell behavior is regulated by transcription factor FoxO1 under hypoxic conditions in developing mouse retinas. Angiogenesis.

[33] Kim YH (2019). A MST1-FOXO1 cascade establishes endothelial tip cell polarity and facilitates sprouting angiogenesis. Nat Commun.

[34] Scallan JP (2021). Foxo1 deletion promotes the growth of new lymphatic valves. J Clin Invest.

[35] Liu P (2021). New soluble angiopoietin analog of Hepta-ANG1 prevents pathological vascular leakage. Biotechnol Bioeng.

[36] Jang C (2009). Angiopoietin-2 exocytosis is stimulated by sphingosine-1-phosphate in human blood and lymphatic endothelial cells. Arterioscler Thromb Vasc Biol.

[37] Marron MB (2007). Regulated proteolytic processing of Tie1 modulates ligand responsiveness of the receptor-tyrosine kinase Tie2. J Biol Chem.

[38] Seegar TC (2010). Tie1-Tie2 interactions mediate functional differences between angiopoietin ligands. Mol Cell.

[39] Maisonpierre PC (1997). Angiopoietin-2, a natural antagonist for Tie2 that disrupts in vivo angiogenesis. Science.

[40] Yuan HT (2009). Angiopoietin 2 is a partial agonist/antagonist of Tie2 signaling in the endothelium. Mol Cell Biol.

[41] Mueller SB, Kontos CD (2016). Tie1: an orphan receptor provides context for angiopoietin-2/Tie2 signaling. J Clin Invest.

[42] Zhang Y (2019). Angiopoietin-Tie signaling pathway in endothelial cells: a computational model. iScience.

[43] Grannemann C (2023). Mechanical activation of lung epithelial cells through the ion channel Piezo1 activates the metalloproteinases ADAM10 and ADAM17 and promotes growth factor and adhesion molecule release. Biomater Adv.

[44] Coste B (2010). Piezo1 and Piezo2 are essential components of distinct mechanically activated cation channels. Science.

[45] Ranade SS (2014). Piezo1, a mechanically activated ion channel, is required for vascular development in mice. Proc Natl Acad Sci U S A.

[46] Oliver G (2020). The lymphatic vasculature in the 21^st^ century: novel functional roles in homeostasis and disease. Cell.

[47] Sabine A (2012). Mechanotransduction, PROX1, and FOXC2 cooperate to control connexin37 and calcineurin during lymphatic-valve formation. Dev Cell.

[48] Andolfo I (2019). *PIEZO1* hypomorphic variants in congenital lymphatic dysplasia cause shape and hydration alterations of red blood cells. Front Physiol.

[49] Bovay E (2018). Multiple roles of lymphatic vessels in peripheral lymph node development. J Exp Med.

[50] Kanady JD (2011). Connexin37 and Connexin43 deficiencies in mice disrupt lymphatic valve development and result in lymphatic disorders including lymphedema and chylothorax. Dev Biol.

[51] Niimi K (2020). FOXO1 regulates developmental lymphangiogenesis by upregulating CXCR4 in the mouse-tail dermis. Development.

[52] Fiedler U (2006). Angiopoietin-2 sensitizes endothelial cells to TNF-alpha and has a crucial role in the induction of inflammation. Nat Med.

[53] Korhonen EA (2016). Tie1 controls angiopoietin function in vascular remodeling and inflammation. J Clin Invest.

[54] McCarthy MJ (1999). Potential roles of metalloprotease mediated ectodomain cleavage in signaling by the endothelial receptor tyrosine kinase Tie-1. Lab Invest.

[55] Caolo V (2020). Shear stress activates ADAM10 sheddase to regulate Notch1 via the Piezo1 force sensor in endothelial cells. Elife.

