# Supplementary material for: The mechanosensory channel PIEZO1 functions upstream of angiopoietin/TIE/FOXO1 signaling in lymphatic development
Source: J Clin Invest. 2024 May 15;134(10):e176577. doi: 10.1172/JCI176577 (PMC11093609; doi:10.1172/JCI176577)
Supplement: Supplemental data [file jci-134-176577-s054.pdf]

The mechanosensory channel PIEZO1 functions upstream of Angiopoietin/TIE/FOXO1 signaling in lymphatic development

Supplemental materials

- Supplemental Figure 1
- Supplemental Figure 2
- Supplemental Figure 3
- Supplemental Figure 4
- Supplemental Figure 5
- Supplemental Figure 6
- Supplemental Figure 7
- Supplemental Figure 8
- Supplemental Figure 9
- Supplemental Figure 10

- Supplemental Table 1
- Supplemental Table 2

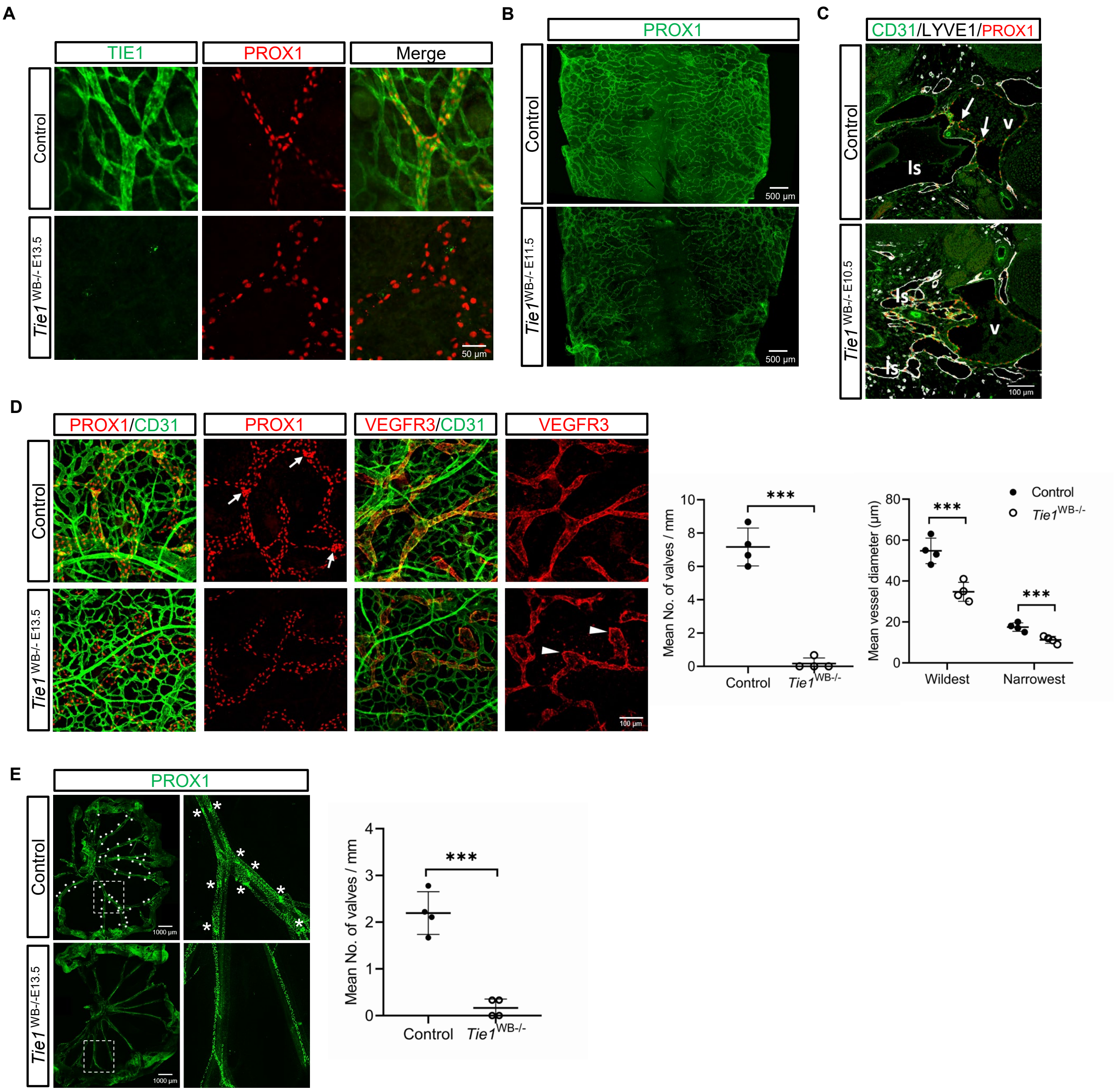

Supplemental Figure 1. *Tie1* gene deletion induces lymphatic vascular and valve abnormalities. (A) Validation of TIE1 deletion through whole-mount E16.5 skin staining with TIE1. **(B)** Reduced lymphangiogenesis observed in the E16.5 dorsal skin of *Tie1*$^{WB-/-E11.5}$ embryos. The dorsal skin, spanning from the armpit to the hind limb, was stained with PROX1. The white line denotes the lymphangiogenic front. **(C)** Frontal sections of E15.5 embryos at the lymph sac region, stained for blood endothelial (CD31) and lymphatic (LYVE1, PROX1) markers. Arrows highlight lymphovenous valves in the control mice. (v: vein, ls: lymph sacs). **(D)** Left panel: Whole-mount skin of E16.5 embryos stained for endothelial and lymphatic markers. Arrows indicate clusters of PROX1^{high} lymphatic valve areas, while arrowheads point to vascular bulge areas in the *Tie1*$^{WB-/-E13.5}$ embryos. Right panel: Quantification of lymphatic valve numbers and vessel diameters in control and *Tie1*$^{WB-/-E13.5}$ embryos, each dot represents the data from one animal. **(E)** Left: Whole-mount mesentery of E18.5 embryos stained with PROX1. PROX1^{high} lymphatic valves are denoted by asterisks in the control, whereas *Tie1*$^{WB-/-E13.5}$ embryos lack lymphatic valves. Right: Quantification of lymphatic valve numbers in mesenteries isolated from control and *Tie1*$^{WB-/-E13.5}$ embryos. each dot represents the data from one animal. Two-tailed, unpaired Student's t-tests were performed to determine the *p* values. Data are expressed as mean ± SD. ***$p < 0.001$.

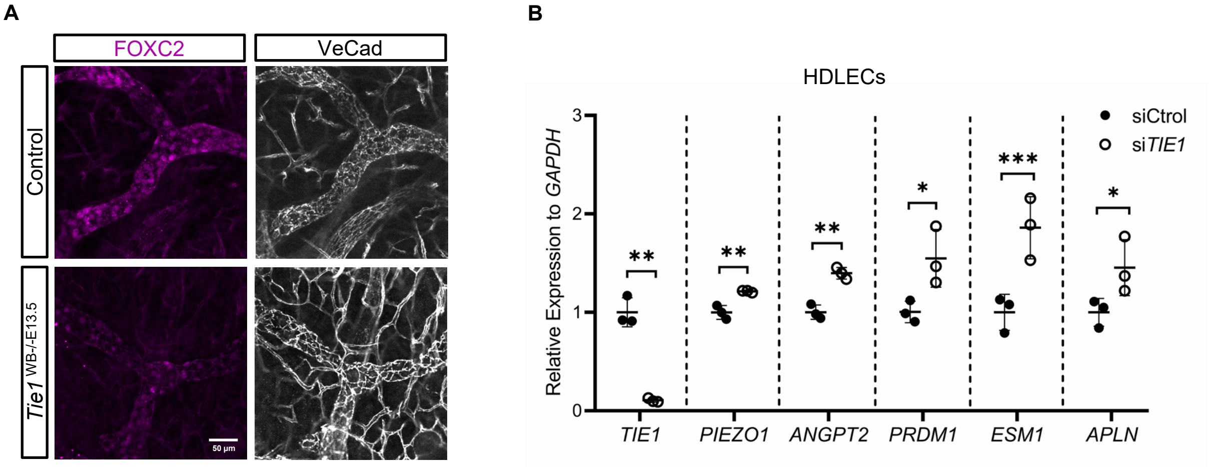

Supplemental Figure 2. Validation of selected genes. (A) E18.5 skin wholemount stained with FOXC2 (left) and VE-Cadherin (right). **(B)** qPCR analysis of selected genes in cDNA generated from HDLEC cells transfected with Control or *TIE1* siRNA. Two-tailed, unpaired Student's t-tests were performed to determine the *p* values. Data are expressed as mean ± SD. *$p < 0.05$, **$p < 0.01$, ***$p < 0.001$.

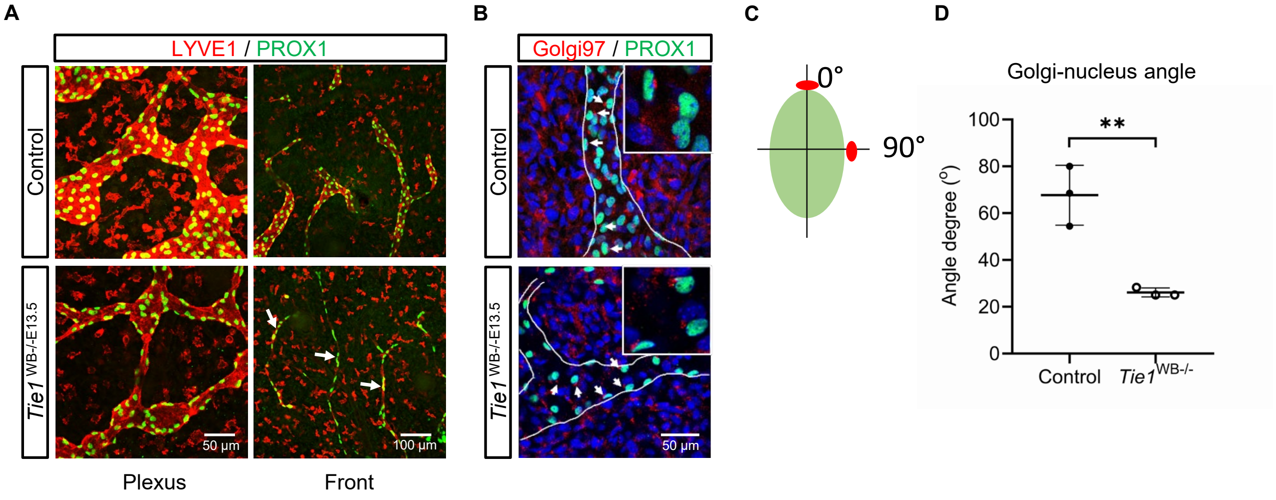

Supplemental Figure 3. Hyperpolarized phenotype of LECs from *Tie1* $^{-/-}$ mice. (A) E16.5 skin wholemount stained with PROX1 and LYVE1 demonstrating very thin unicellular extensions at the lymphangiogenic front. **(B)** E16.5 skin wholemount stained with PROX1 and Golgi97. **(C)** Schematic demonstrating definition of the Golgi-nucleus angle. When the Golgi apparatus is on the longitudinal axis of the nuclei the angle is 0. When it is on the horizontal axis of the nuclei the angle is 90. **(D)** Quantification of Golgi-nucleus angle. 4 images were counted from each mouse. Each dot represents data from a single mouse. Two-tailed, unpaired Student's t-tests were performed to determine the *p* values. Data are expressed as mean ± SD. $^{**}p < 0.01$.

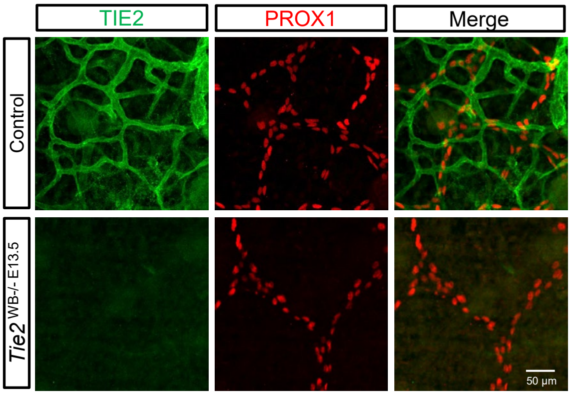

Supplemental Figure 4. Validation of TIE2 deletion in whole-mount E16.5 skin samples from *Tie2*$^{WB\text{-}/\text{-}E13.5}$ embryo.

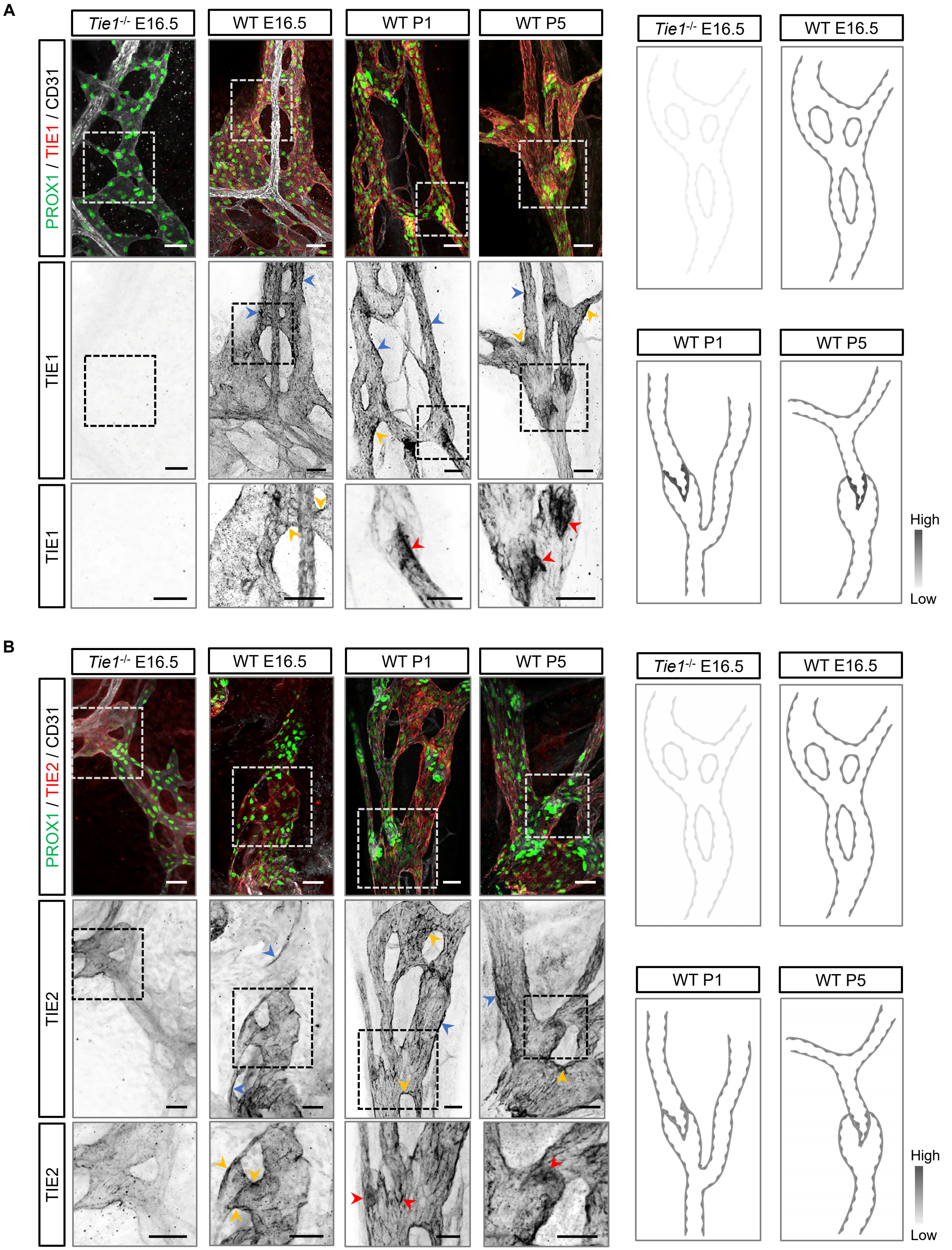

Supplemental Figure 5. TIE receptors expression pattern in mesentery lymphatic vessels at different developmental stages. (A) TIE1 expression pattern in *Tie1*$^{-/-}$ E16.5, WT E16.5, WT P1 and WT P5 mesenteries. **(B)** TIE2 expression pattern in *Tie1*$^{-/-}$ E16.5, WT E16.5, WT P1 and WT P5 mesenteries. Arrow heads point to TIE receptor expressed in branching (yellow), non-branching (blue) or valve (red) areas. Scale bar: 50 µm.

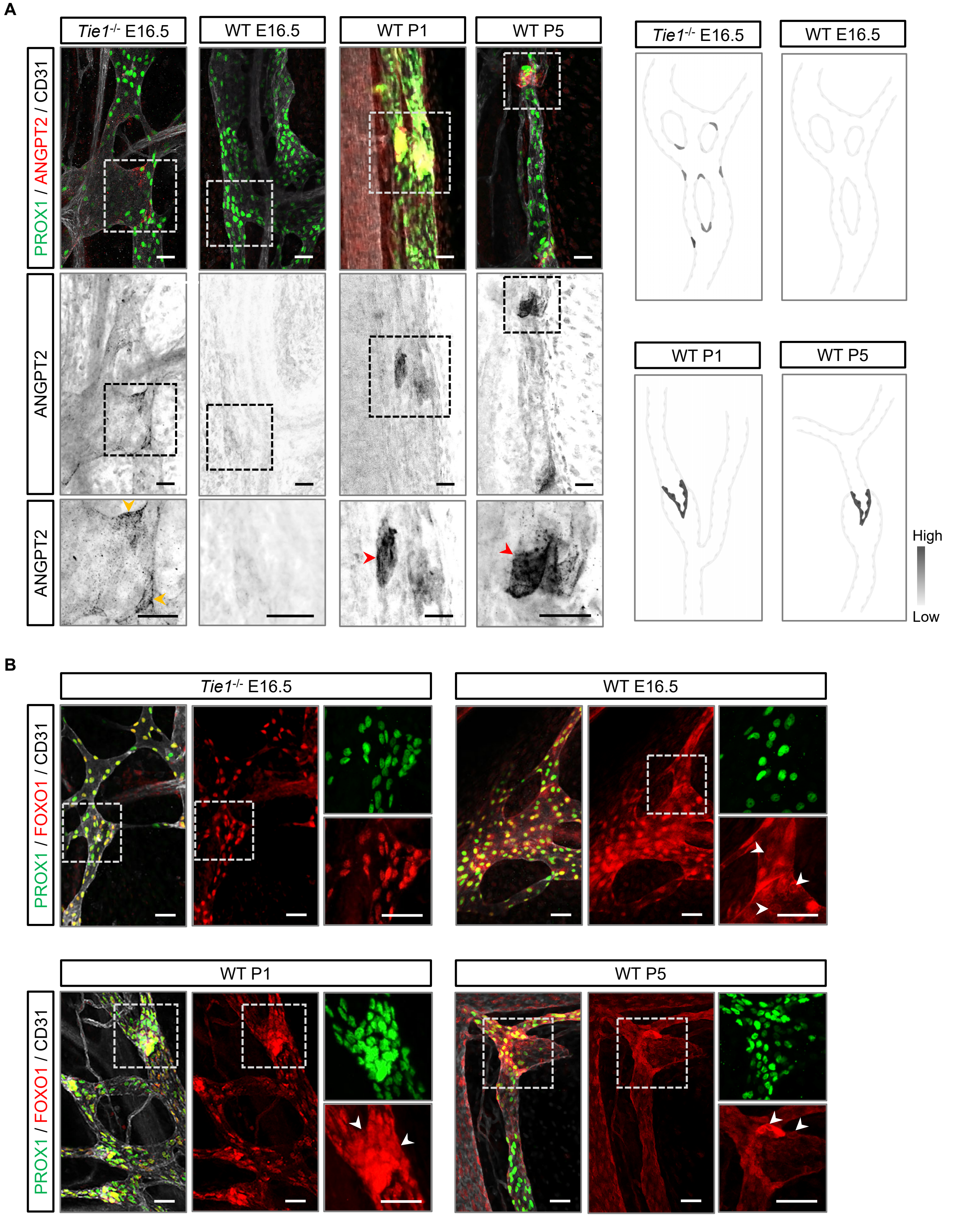

Supplemental Figure 6. (A) Angiopoietin 2 expression pattern in *Tie1*$^{-/-}$ E16.5, WT E16.5, WT P1 and WT P5 mesenteries. Arrow heads point to ANGPT2 high-expression areas. **(B)** Cellular localization of FOXO1 in *Tie1*$^{-/-}$ E16.5, WT E16.5, WT P1 and WT P5 mesenteries. Arrow heads point to cells with cytoplasmic or cytoplasmic plus nuclear FOXO1 localization. Scale bar: 50 µm.

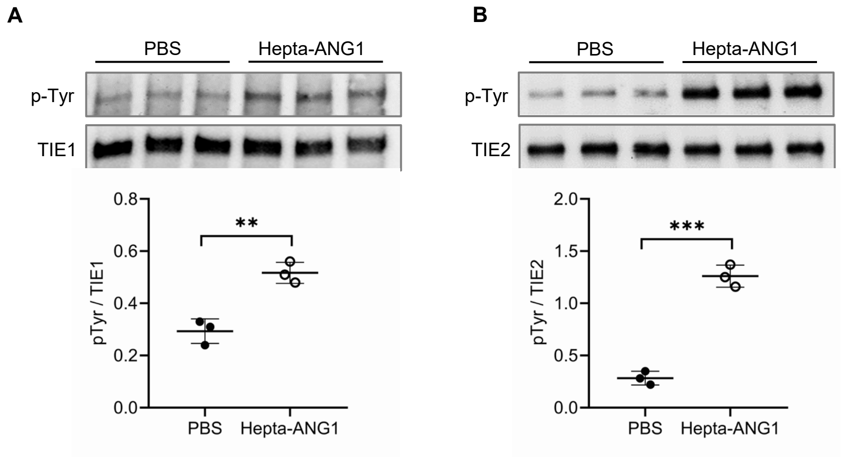

Supplemental Figure 7. TIE activation by Hepta-ANG1. Western blot analysis of TIE1 **(A)** and TIE2 **(B)** phosphorylation from HDLECs treated with PBS or Hepta-ANG1 (1 µg/ml) for 30 min. Each band represent a biological replicate sample (n=3). Two-tailed, unpaired Student's t-tests were performed to determine the *p* values. Data are expressed as mean ± SD. $^{**}p < 0.01$, $^{***}p < 0.001$.

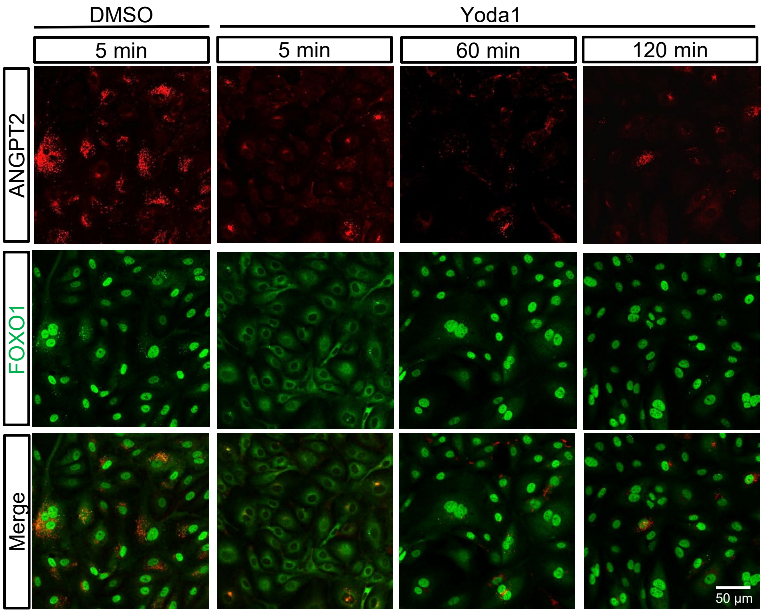

Supplemental Figure 8. FOXO1 shuttling induced by Yoda1 is dynamic and reversible. Serum-starved HDLECs were treated with either DMSO or Yoda1 for a duration of 30 minutes, followed by two PBS washes, and then maintained in Endothelial Cell Basal Medium MV2. At specified time points, the cells were fixed and examined for signals related to ANGPT2 and FOXO1.

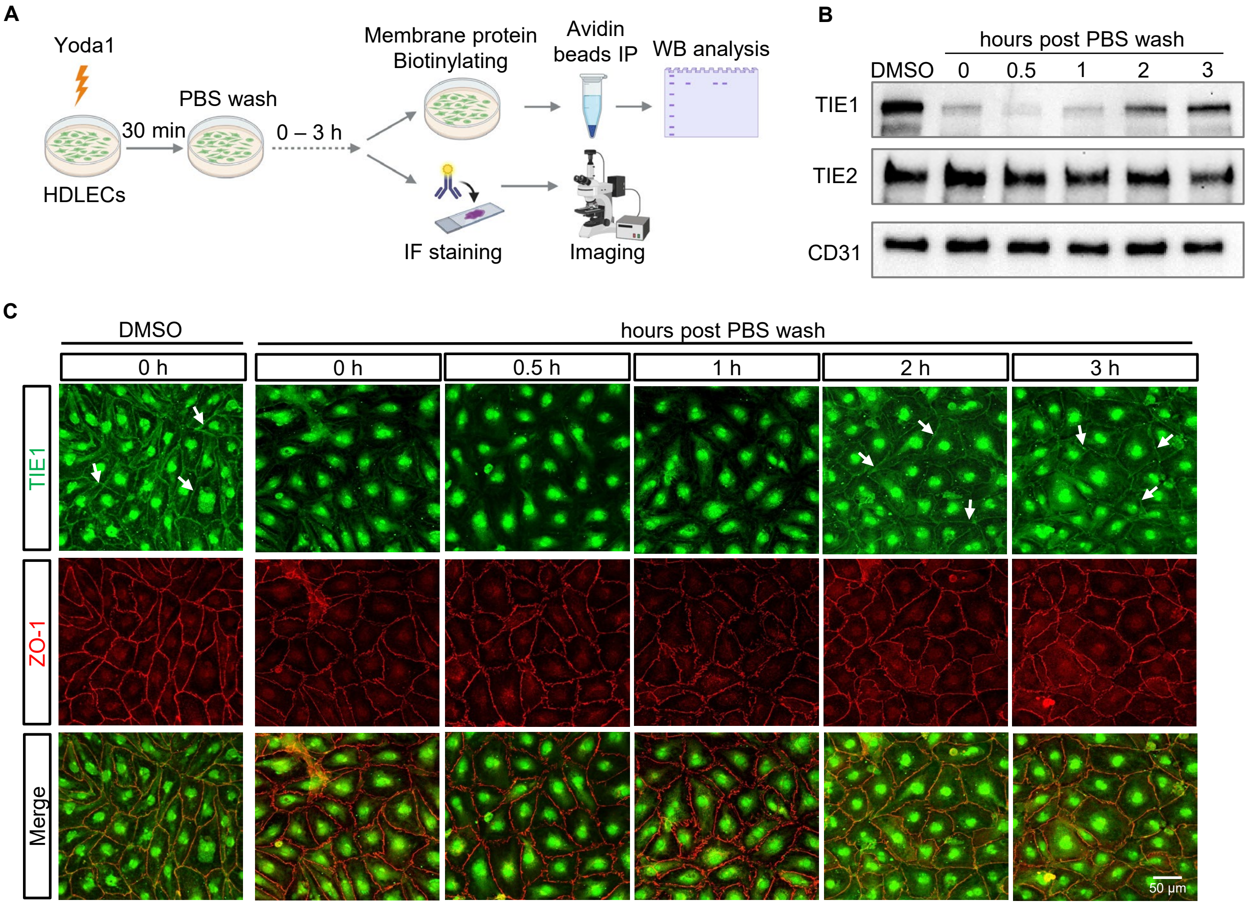

Supplemental Figure 9. Rapid re-accumulation of TIE1 on the surface of HDLECs following Yoda1-induced shedding. (A) Experimental design schematic: HDLECs were exposed to Yoda1 for 30 minutes, then washed three time with PBS. Subsequently, cells were incubated in serum-free media for durations ranging from 0 to 3 hours. Cell surface proteins were biotinylated for isolation and analyzed via Western blot. Concurrently, cells underwent immunofluorescence staining for TIE1 and ZO-1. DMSO-treated cells served as the control. **(B)** Western blot analysis: membrane-bound TIE1 and TIE2 levels were assessed, with CD31 as the loading control. Notably, TIE1 re-accumulation on the plasma membrane was detectable within 2 hours post-Yoda1 washout. Each band represent a biological replicate sample (n=3). **(C)** Immunofluorescence staining: staining for TIE1 and ZO-1 was performed. The arrows indicate the localization of TIE1 at the cell-cell junctions.

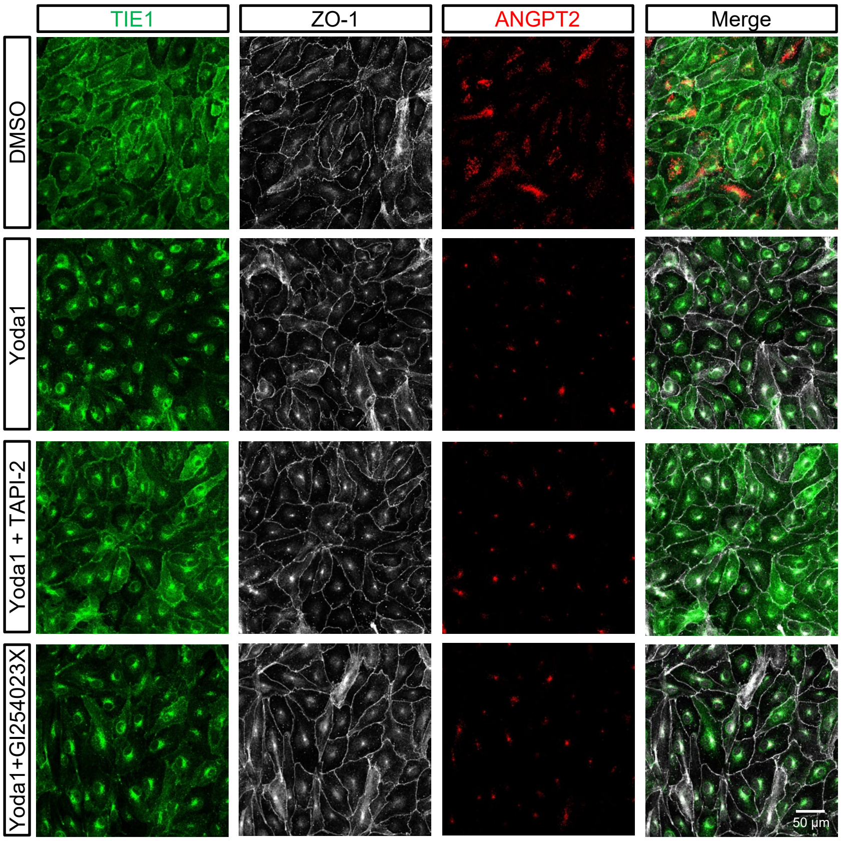

Supplemental Figure 10. Yoda1 induced TIE1 shedding was blunted by MMP/ADAM17 inhibitor TAPI-2. HDLECs were pre-treated with vehicle, MMP/ADAM17 inhibitor TAPI-2 or ADAM10 inhibitor GI254023X followed by Yoda1 treatment. TIE1 immunostaining indicated the Yoda1 induced TIE1 shedding was largely blunted in TAPI-2 but not GI254023X pretreated group.

Supplemental Table 1. qPCR primers

Mouse *Tie1*-F	GCATGAAACTTCGCAAGGCCA
Mouse *Tie1*-R	GGAGTCGAGGTGCAGTCAAA
Human *FOXC2*-F	ACTCCTACGACTGCACGAAATACTG
Human *FOXC2*-R	GTCTCTGCAGCCCCTTAATTGT
Human *GATA2*-F	GCGTCTCCAGCCTCATCTTCCGC
Human *GATA2*-R	CGAGTCTTGCTGCGCCTGCTT
Human *GJA4*-F	ACACCCACCCTGGTCTACC
Human *GJA4*-R	CACTGGCGACATAGGTGCC
Human *ITGA9*-F	CGGAATCATGTCTCCAACCT
Human *ITGA9*-R	TCTCTGCACCACCAGATGAG
Human *PRDM1*-F	AGAGAAAGAAGAAACTGGCCTGAAA
Human *PRDM1*-R	ATCTGATGACTCATAAAGGCTGCAC
Human *PIEZO1*-F	CAGGCCTATGAGGAGCTGTC
Human *PIEZO1*-R	TTGTAGAGCTCCCGCTTCAT
Human *ANGPT2*-F	TGCAAATGTTCACAAATGCTAA
Human *ANGPT2*-R	AAGTTGGAAGGACCACATGC
Human *ESM1*-F	ACTTGCTACCGCACAGTCTCAG
Human *ESM1*-R	AATCCATCCCGAAGGTGCCGTA
Human *APLN*-F	CCAGAGGGTCAAGGAATGGGC
Human *APLN*-R	ATAACCGCCGGGGGTGGGCA
Human *FOXO1*	Ordered from Santa Cruz (sc-35382-PR)
Human *TIE1*	Ordered from Santa Cruz (sc-36675-PR)

Supplemental Table 2. Antibodies

Antibody	Host	Supplier	Number	Dilution
PROX1	Goat	R&D	AF2727	1:200 (IF)
PROX1	Rabbit	AngioBio	11-002P	1:200 (IF)
TIE1	Goat	R&D	AF619	1:200 (IF) or 1:1000 (WB)
hTIE2	Goat	R&D	AF313	1:1000 (WB)
mTIE2	Goat	R&D	AF762	1:200 (IF)
mCD31	Rat	BD	553370	1:100 (IF)
mLYVE1	Goat	R&D	AF2125	1:200 (IF)
mLYVE1	Rabbit	AngioBio	11-034	1:200(IF)
mVEGFR3	Goat	R&D	AF743	1:200 (IF)
α-Tubulin	Mouse	Santa Cruz	Sc-32293	1:1000 (WB)
FOXO1	Rabbit	Cell Signaling	C29H4	1:200 (tissue) or 1:500 (cell)
FOXC2	Sheep	R&D	AF6989	1:200 (IF)
mVE-Cadherin	Rat	BD	555289	1:100 (IF)
Golgi97	Rabbit	Cell Signaling	13192S	1:100 (IF)
ANGPT2	Human	Gift from Dr.Koh		1:250 (IF)
p-AKT(S473)	Rabbit	Cell Signaling	4060s	1:1000 (WB)
panAKT	Rabbit	Cell Signaling	4691s	1:1000 (WB)
pTyrosine	Mouse	Millipore	05-321	1:1000(WB)
LYVE1-APC	Rat	R&D	FAB2125A	1:10 (Flow)
CD31-PECy7	Rat	Invitrogen	25-0311-82	1:50 (Flow)
CD45-PE	Rat	Invitrogen	12-0451-82	1:100 (Flow)
ZO-1	Rabbit	Abcam	ab216880	1:200 (IF)
